# Environmental, genetic and physiological factors governing potato endodormancy: from tuber development to endodormancy break

**DOI:** 10.3389/fpls.2026.1858517

**Published:** 2026-10-12

**Authors:** Fabian Villamil-Bolaños, Sofia Kourmpetli, Edwin van der Vossen, Gemma Chope, Katherine Cools, Ron van der Hulst, Ernst-Jan Eggers, M. Carmen Alamar

**Affiliations:** 1 Postharvest Research Group, Cranfield University, Cranfield, United Kingdom; 2 Centre for Soil, Agrifood and Biosciences, Cranfield University, Cranfield, United Kingdom; 3 Solynta, Wageningen, Netherlands; 4 Agricultural Science R&D, PepsiCo International Limited, Leicester, United Kingdom; 5 Cools Agriscience Consultancy Limited, Crowthorne, United Kingdom

**Keywords:** cell cycle, plant growth regulators, postharvest storage, preharvest, quantitative trait loci (QTL), *Solanum tuberosum* L., sprouting

## Abstract

Potato tuber dormancy and sprouting are complex traits with major implications for storage, quality, and postharvest management. Historically, these traits were controlled using chlorpropham (CIPC); however, following its ban in 2019 in the European Union, research aimed at understanding and managing dormancy and sprouting has increased substantially. Integrating findings across studies remains challenging due to the complexity of these traits, gaps in current knowledge, and differences in phenotyping approaches. In this review, we critically examine the challenges associated with evaluating dormancy and dormancy break progression and the current state of the knowledge across key developmental stages, from tuber formation, dormancy maintenance, to dormancy break. During tuber formation, we explore environmental signals that influence tuber induction and may contribute to dormancy extension, the reallocation of sugars from leaves to stolons, and the crosstalk amongst regulatory compounds. For dormancy maintenance, we analyse mechanisms of cell cycle arrest and highlight the roles of plant growth regulators in maintaining quiescence. Dormancy break is discussed in relation to sugar metabolism, symplastic reconnection, cell cycle reactivation, reactive oxygen species dynamics, and the influence of gibberellins and cytokinins. We also provide an overview of quantitative trait loci (QTLs) associated with dormancy and sprouting identified across multiple studies. By structuring this synthesis around developmental stages rather than individual processes, we identify critical research gaps to support future research aimed at improving dormancy control in potatoes.

## 1 Introduction

Potato (*Solanum tuberosum*) ranks sixth globally in terms of agricultural output, surpassed only by sugarcane, maize, rice, wheat, and oil palm. The average annual worldwide potato production is estimated to be 376 million tons, whilst the export and import value totalled more than 9 billion USD (FAOSTAT, 2023). Potatoes are an important crop used for human consumption, and animal feed, with around two-thirds of the global potato yield destined to feed approximately 1.3 billion people (Devaux et al., 2021). However, up to 25% of potato production is lost during storage, largely due to dormancy break and subsequent sprouting (Thoma and Zheljazkov, 2022). Potato sprouting has been historically controlled in store using CIPC (chlorpropham) because of its low cost and high efficacy (Visse-Mansiaux et al., 2021). However, following the EU ban in 2019 due to health and safety concerns (European Commission, 2019), alternative sprout control strategies have become necessary. For this reason, understanding and controlling both pre- and postharvest mechanisms underlying potato dormancy is essential, as this knowledge will enable the development of sustainable strategies ranging from gene editing to agricultural practices, and lowering costs associated with postharvest tuber storage and management.

Potato tubers are underground storage structures that develop from stolons. These stolons produce small nodes that thicken and store starch and other nutrients, eventually forming tubers. Small buds on the tuber surface have the potential to develop into new sprouts (Stark et al., 2020). It is thought that these buds become dormant during tuber development (Claassens and Vreugdenhil, 2000) and remain dormant for an undetermined period after the tuber has fully developed (Di et al., 2024). As such, dormancy refers to the absence of bud growth, whilst dormancy break marks the transition from suppressed growth to renewed activity. This transition coincides with a cascade of metabolic changes during which the tuber shifts from sink to source, providing assimilates for the resumption of bud growth. These changes can lead to significant crop losses due to reduced quality of raw material manifested as lower firmness, disrupted sugar metabolism, and greater susceptibility to pathogens and postharvest damage (Alamar et al., 2017; Sonnewald and Sonnewald, 2014). Therefore, the control and management of potato dormancy are crucial for the potato industry.

Lang et al. (1987) described three phases of dormancy in stem meristems that have been also applied to tuber meristems; endodormancy, ecodormancy and paradormancy. Endodormancy is controlled by internal physiological factors, preventing bud growth even under favourable conditions; ecodormancy occurs when external factors, such as low temperatures or drought, delay dormancy break; and paradormancy is driven by apical dominance and hormonal signals, mainly auxins, which suppress lateral bud growth. However, a recent review suggests refining the definition of ecodormancy to differentiate between tubers stored for the processing industry (8-10 °C) and those stored for seed purposes (3-4 °C) (Cools and Chope, 2026).

Understanding potato tuber endodormancy remains challenging due to several factors. First, endodormancy is a complex trait regulated by interconnected hormonal, genetic, metabolic, and environmental networks, whose interactions are not yet fully understood. This complexity is further increased by the autotetraploid nature of most commercial potato cultivars, which complicates genetic analyses and the translation of findings from diploid systems. Second, significant gaps remain in our mechanistic understanding of endodormancy establishment, maintenance, and break. A third challenge is the lack of standardised phenotyping protocols and consensus terminology, which hinders comparisons amongst studies and the consistent classification of dormancy stages. Therefore, this review aims to integrate current knowledge within a chronological framework spanning tuber development, endodormancy maintenance, endodormancy break, and sprouting, highlighting how internal physiological and environmental factors interact throughout these stages, whilst also emphasising discrepancies amongst studies arising from differences in phenotyping protocols.

## 2 Endodormancy, endodormancy break, and sprouting: challenges in definition and phenotyping

One of the major challenges in potato endodormancy research is the lack of consensus regarding the definition and phenotyping of endodormancy, endodormancy break, and sprouting. Although these terms are frequently used interchangeably, they represent distinct biological stages. Endodormancy is characterised by the temporary arrest of bud growth, whereas sprouting corresponds to the subsequent phase of active bud elongation. Endodormancy break is typically defined when buds reach 2 mm in length, although thresholds of 1 mm and 3 mm have also been used (Chiab et al., 2023; Gautam et al., 2024; Hou et al., 2019). However, these phenotypic markers are more closely associated with the onset of sprout growth, making it difficult to clearly distinguish endodormancy break from early sprouting. This distinction is highly relevant because endodormancy break and sprout growth represent different traits. Whilst some genotypes may break endodormancy at similar times, they can differ substantially in subsequent sprout elongation rates. As a result, phenotyping based exclusively on sprout length may reflect variation in sprout growth rather than endodormancy break itself. On the other hand, other studies have assessed postharvest tuber behaviour using alternative criteria, including days to sprouting (Freyre et al., 1994), percentage of sprouted tubers (Pasare et al., 2013), time to 50% sprouting (Danieli et al., 2023), sprout length (Sharma et al., 2021), and qualitative developmental stages such as peeping (Foukaraki et al., 2016b) and budding (Zheng et al., 2024). However, these latter terms lack standardised definitions and generally refer to the first visible signs of bud emergence, making comparisons amongst studies difficult.

This conceptual ambiguity is further complicated by the inconsistent use of terms such as endodormancy, paradormancy and ecodormancy. Recent work has questioned whether the classical endodormancy-ecodormancy framework adequately describes potato tuber dormancy under commercial storage conditions. Cools and Chope (2026) proposed distinguishing growth suppression under cool storage (store quiescence) from responses to colder temperatures (chilling response), highlighting the lack of consensus surrounding postharvest tuber terminology. Commercial storage practices exacerbates this issue, as tubers may be subjected to wound-healing periods, immediate cold storage, or prolonged storage before evaluation. Consequently, differences in storage history can substantially influence physiological status and hinder comparisons amongst studies.

Throughout this review, the terminology and phenotypic thresholds used in the original studies are reported. This approach aims to highlight that differences in metabolic, physiological, and molecular responses amongst studies may not necessarily reflect biological inconsistencies but may instead arise from the evaluation of distinct postharvest processes (e.g., endodormancy break versus sprouting) or from differences in storage conditions and phenotyping methodologies.

## 3 Genotype-environment interactions in potato endodormancy and sprouting

Plant traits are not solely determined by genetics, but rather from a dynamic interaction between genotype (G), and environment (E) (including management practices). This G × E interaction plays a critical role in shaping complex traits and influencing their stability under diverse growing conditions. The genotype refers to the complete coding sequences, and regulatory elements, which collectively determine its hereditary characteristics. However, these characteristics are usually not dictated by individual alleles but by complex allelic interactions and their regulation in response to environmental cues. The environment does then modulate biological traits, affecting processes such as gene expression, cellular differentiation, and development (Ibañez et al., 2021).

Genetic factors influence the duration of endodormancy and sprouting. In the population derived from diploid potato groups *Phureja* and *Tuberosum* analysed by Sharma et al. (2021), broad-sense heritability (H²) values for sprouting ranged from 0.70 to 0.90, reflecting highly stable genetic effects across years and time-points. More recently, Vexler et al. (2024) also observed high marker-based H² (0.749) for sprouting in a diverse diploid breeding panel. However, it is worth noting that the specific postharvest storage conditions were not described and likely varied across participating breeding programmes. Earlier work by Thompson et al. (1980), used a diploid interpopulation hybrid derived from *S. phureja* and *S. stenotomum*, and reported an H² of 0.57 for sprouting, evaluated as the time when a sprout reached 2 mm under controlled postharvest conditions (21 °C). Whilst previous studies were largely conducted in diploid populations, Jiao et al. (2026) evaluated tetraploid *S. tuberosum* germplasm field locations and two storage conditions (4 °C and 18 °C) and reported H^2^ estimates for tuber dormancy ranging from 0.64 to 0.86. This aligns with the results reported by Visse-Mansiaux et al. (2022), whose dataset was composed predominantly of tetraploid commercial cultivars. In their study, the variety effect (G) explained the largest proportion of sprouting variation (60.3%), followed by the environmental components (E) (year [13.9%] and location [5.4%]). Together, these findings suggest that genotype plays a major role in controlling sprouting, nevertheless the environment and G × E interactions remain important. In Jiao et al. (2026), despite the high H^2^, the proportion of phenotypic variation explained by genotype decreased from 59.7% at 18 °C storage to 22.6% at 4 °C, where location (27.7%) and genotype x location interaction (20.0%) became major sources of variation. This highlights the continued importance of environmental management for controlling dormancy and sprouting.

However, the genetic and physiological factors governing endodormancy and sprouting are highly complex and difficult to disentangle. Whilst, historically, the potato industry has relied on chemical inhibitors such as CIPC to prevent premature sprouting, a deeper understanding of how preharvest environmental factors regulate endodormancy is essential for developing more sustainable solutions. Despite its importance, relatively few studies have investigated the impact of preharvest environmental cues on postharvest endodormancy regulation. Therefore, the following sections explore environmental factors regulating potato dormancy from tuber formation through to dormancy break and highlight key research gaps to elucidate the underlying mechanisms of dormancy.

## 4 Tuber formation

Tuber formation, or tuberisation, is the developmental process by which stolons transition into storage organs through coordinated changes in growth, carbon allocation, starch accumulation, and storage-protein deposition. Although tuberisation is usually described as a sequence of developmental stages, the boundaries between these stages are not always clearly defined. We identify three main stages: (I) tuber induction, in which environmental signals are perceived and initiate tuberisation; (II) tuber initiation, when stolon tissues undergo metabolic and developmental changes leading to tuber formation; and (III) tuber bulking, during which tubers enlarge through cell expansion and reserve accumulation.

The relationship between tuberisation and the onset of endodormancy remains unresolved. Some studies suggest that buds enter endodormancy as tubers begin to develop (Claassens and Vreugdenhil, 2000), whereas others propose that a period of bud development is required before the tissue reaches a physiological state capable of entering endodormancy (Cools and Chope, 2026). This distinction is important because environmental and metabolic conditions during tuber formation may influence not only tuber development, but also subsequent dormancy duration and sprouting behaviour. Further research is therefore needed to clarify when endodormancy is established and which physiological mechanisms regulate its onset.

This section examines tuber formation as a potential determinant of dormancy behaviour. It first considers environmental cues that regulate tuberisation and may influence subsequent endodormancy and sprouting, before reviewing metabolic shifts associated with tuber induction and initiation.

### 4.1 Environmental factors regulating tuberisation and endodormancy

Preharvest factors, including soil temperature, nutrient availability (e.g., nitrogen) and photoperiod have been reported to regulate tuberisation (Dutta et al., 2024; Hastilestari et al., 2018; Mathura et al., 2024; Zhang et al., 2024). Their impact on both tuberisation and endodormancy is discussed below and summarised in **Figure 1**.

**Figure 1:**
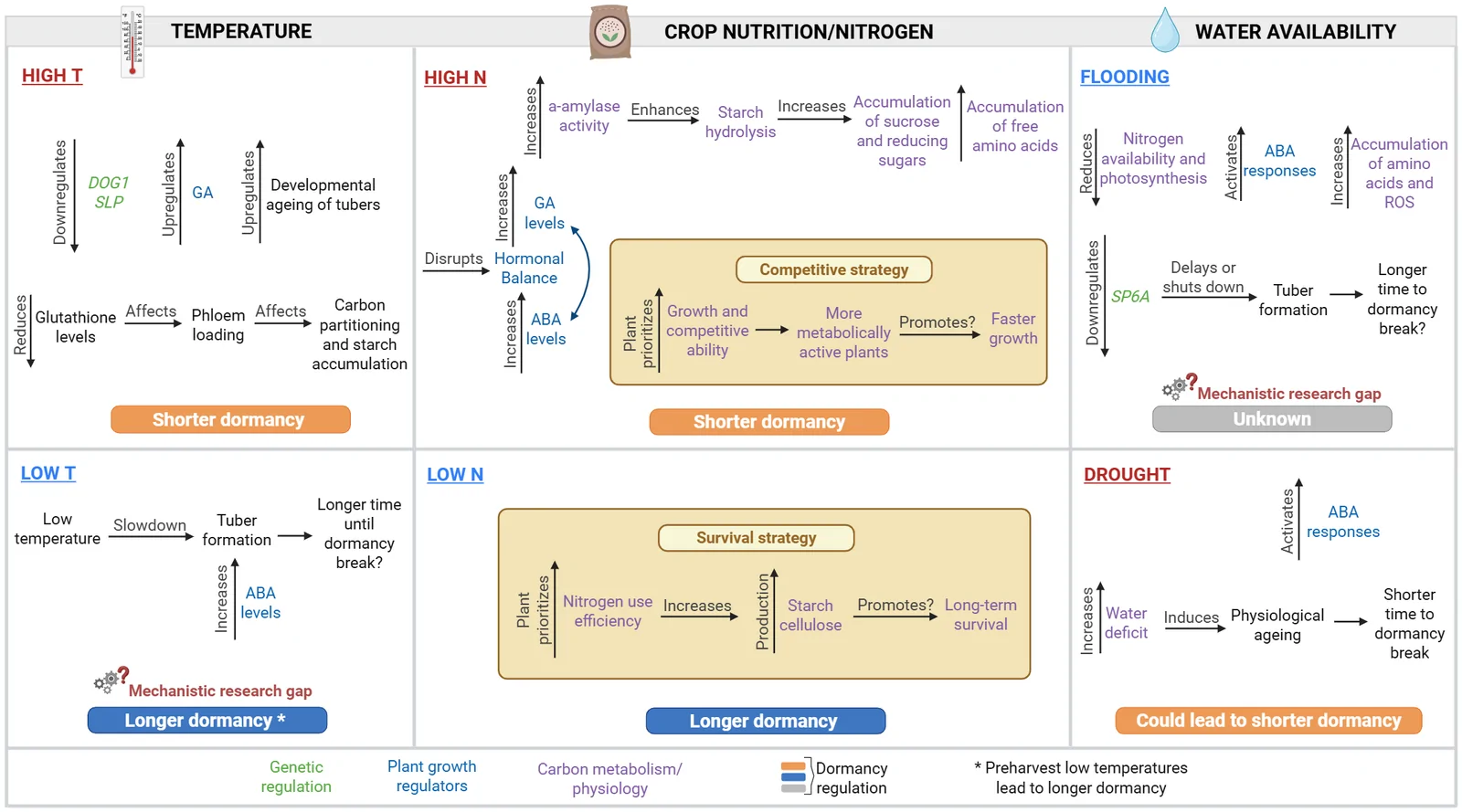
Environmental conditions during crop establishment and growth, including temperature, nitrogen supply, and water availability, induce specific metabolic and physiological responses in tubers. These preharvest signals influence carbon metabolism, hormonal balance, and stress responses, ultimately shaping postharvest dormancy behaviour and extension.

#### 4.1.1 Temperature during tuber development

With the growing pressure caused by global warming, potato production will face several challenges associated with rising temperatures. It has been reported that temperatures between 14–22 °C are optimal for tuber formation and yield (Hancock et al., 2014), whilst temperatures above 25 °C disrupt biomass partitioning and reduce the weight of developing tubers in *S. tuberosum* cv. Agria and Désirée (Hastilestari et al., 2018; Zagorščak et al., 2025). However, the role of high temperatures during tuberisation on postharvest tuber endodormancy has only recently gained attention. Recent evidence indicates that genotype-dependent differences exist for traits such as heat tolerance, heat-induced sprout formation during tuber development, and postharvest endodormancy duration. Studies from the Texas A&M Potato Breeding Program (Gautam et al., 2024) found that exposure to extreme temperatures during plant growth (35/25 °C day/night) accelerated sprouting initiation (assessed at 20 °C when sprout length reached 2 mm), compromising tuber quality and storability. Cultivar-dependent differences were also observed: cv. Vanguard Russet exhibited greater heat tolerance and consistently longer time to sprouting, whereas cv. Russet Burbank and cv. Atlantic were more heat-sensitive and displayed shorter time to sprouting under heat stress conditions. Similarly, Zhang et al. (2021) evaluated both heat-induced sprouting during tuber development and postharvest tuber endodormancy (assessed at 21 °C when sprout length reached 2 mm). They reported substantial genotypic variation, with cvs. Eramosa and Superior showing pronounced heat-induced sprouting, whilst others, such as cv. Innovator, exhibiting shorter time to sprouting, or no heat-induced sprouting.

In addition to these observations, high temperatures have been associated with changes indicative of accelerated physiological ageing. For example, compared with immature tubers maintained under ambient conditions, heat-exposed immature tubers displayed transcriptomic profiles that clustered more closely with those of mature tubers, suggesting accelerated maturation (Guillemette et al., 2025), and a shift towards a more advanced developmental stage that may increase the likelihood of early sprouting during storage. Moreover, heat stress can impair carbon partitioning processes, as reductions in leaf glutathione levels under elevated temperatures may affect the redox regulation of sucrose transporters involved in phloem loading, thereby limiting starch accumulation in developing stolons (Hancock et al., 2014). At the molecular level, heat stress has been suggested to be associated with reduced expression of dormancy-related genes, including *Delay of Germination 1* (*DOG1*) and the *protease gene SLP* (both typically expressed during the dormant state), alongside enrichment of gibberellin-related biosynthesis pathways in potato tubers (Zhang et al., 2021). These transcriptional responses resemble those reported during seed dormancy break in *Arabidopsis thaliana* (Bentsink et al., 2006). Although, the underlying regulatory mechanisms have been established in *A. thaliana*, whereas their roles in potato remain inferred from transcriptomic associations and require functional validation.

Together, these findings support strong G × E interactions and suggest that elevated preharvest temperatures may shorten postharvest endodormancy in some genotypes, possibly through accelerated physiological ageing and overlap between heat-induced and sprouting-related pathways. Further research should investigate the physiological and molecular mechanisms underlying this response, particularly the extent to which conditions during tuberisation influence subsequent endodormancy duration and postharvest storability.

#### 4.1.2 Role of nitrogen

Nitrogen (N) plays a role in potato tuberisation and overall plant development. High N availability has been associated with delayed tuber initiation, reduced tuber number, and increased tuber weight (Aksenova et al., 2012; Zakaria et al., 2007; Zhang et al., 2022). Nitrogen availability has also been shown to influence sprouting. Reduced N application can delay postharvest sprouting by 3–6 weeks, whereas higher N levels accelerated sprout emergence timing in cvs. Eshu3, Zshu5, and Hshu3 (assessed at 25 °C when sprout length reached 2 mm) (Zhang et al., 2022). This effect is mediated through hormonal regulation. Tubers from N-treated plants exhibited significantly higher levels of gibberellic acid (GA_1_) in the apical bud, up to 6-fold higher, whilst abscisic acid (ABA), a key dormancy-maintaining hormone, increased to a lesser extent (2.6-fold). This N-induced change in hormonal balance may contribute to the formation of physiologically immature tubers (Zhang et al., 2020a), potentially resulting in shorter endodormancy periods, and increased susceptibility to sprouting and quality deterioration during storage.

Carbohydrate metabolism, which is closely linked to dormancy status and subsequent sprout growth, is also affected by N application. Zhang et al. (2020a) reported that increased N availability enhanced α-amylase activity and promoted starch hydrolysis, leading to higher concentrations of sucrose and reducing sugars at harvest and during cold storage (4–5 °C). These soluble sugars may provide readily available carbon to support bud activation and early sprout growth and could therefore contribute to shorter endodormancy. However, because these responses were observed under cold-storage conditions, they may reflect changes associated with cold-induced sweetening rather than direct regulation of endodormancy. Further work under conditions that distinguish endodormancy break from ecodormancy release is needed to clarify this relationship.

The precise mechanism by which N regulates both tuberisation and dormancy remains unclear. We propose that its role can be interpreted within the framework of the carbon:nitrogen (C:N) ratio. Initially described in forest systems, plants under low N adopt a survival strategy by prioritising N use efficiency, whereas under adequate or N-rich conditions, plants prioritise growth and compete with other plants for carbon assimilation through photosynthesis (Zhang et al., 2019; Zheng et al., 2018). This suggests that under low N availability, plants may allocate resources towards the development of structural compounds such as cellulose or starch and extend endodormancy periods to ensure survival. Conversely, under N-rich conditions, plants are more metabolically active, and endodormancy may be shortened to prioritise growth. Extrapolating these observations to tubers, those produced under adequate or N-rich conditions may exhibit greater metabolic activity owing to increased synthesis of compounds that support growth resumption. In contrast, tubers produced under low N conditions may prioritise the accumulation of structural compounds, potentially contributing to prolonged endodormancy and improved resilience to adverse conditions.

In addition to N rate, the form of N has also been found to influence *S. tuberosum* (cv. Kexin-1 and cv Shepody) plant growth dynamics. Nitrate has been shown to promote earlier tuber formation compared to ammonium (Gao et al., 2014). Similarly, using cvs. Zhongshu 18 and Shepody, Ye et al. (2024) demonstrated that canopy development differs depending on N form, with ammonium nutrition extending canopy duration and maintaining green leaf area beyond the optimal harvest window. This prolonged vegetative growth delays physiological maturity, postponing tuber bulking, and skin set. These developmental effects are particularly relevant because tuber physiological maturity at harvest may be associated with endodormancy duration. Delayed physiological maturity often results in immature tubers at harvest, which may be more prone to late sprouting during storage. However, whilst the effects of N rate on endodormancy are relatively well documented, the role of N form remains poorly understood. Evidence from other plant systems suggests a potential link: nitrate accumulation led to shorter seed dormancy in *A. thaliana* (Alboresi et al., 2005), whereas ammonium has not been implicated in endodormancy regulation in this species. However, no studies have directly examined how different N forms influence potato tuber endodormancy. Given the strong effects of N form on tuberisation and plant development, this represents an important knowledge gap requiring further investigation.

#### 4.1.3 Water availability

Despite increasing concerns over global warming, declining water resources, and the substantial research on water and irrigation management, studies on the role of water availability during the growing season in regulating potato endodormancy remain scarce. A recent multi-omics study found that flooding-induced root hypoxia impairs root conductance and water uptake, triggering drought-like responses in the shoot, including ABA and ethylene signalling, whilst reducing photosynthetic performance and tuber sink strength. Flooding induced a transient increase in the tuberigen SP6A, suggesting promotion of tuber initiation; however, prolonged flooding was associated with SP6A downregulation and inhibition of the tuberisation process (Zagorščak et al., 2025). The effect of flooding on endodormancy release remains unknown.

In contrast, a study by Li et al. (2023) focusing on tuberisation found that water deficit significantly reduces tuber dry matter content. This reduction, together with stress-induced accelerated physiological aging, may contribute to shorter endodormancy periods. Direct evidence linking water availability during growth to endodormancy regulation remains limited and warrants further research.

### 4.2 From leaves to stolons: environmental signals induce tuberisation

Tuberisation is thought to have evolved as a response to fluctuating climates, enabling potato plants to store carbohydrates in tubers during periods of adverse environmental conditions. In the native environment of potatoes, the equatorial highlands of South America, tuberisation is primarily regulated by low ambient temperatures, hormonal signalling, and stress-related cues under consistent 12-hour day lengths (similar to short-day [SD] conditions) (Bou-Torrent et al., 2011; Rodríguez-Falcón et al., 2006). In contrast, in modern cultivated potatoes, resulting from the genetic mixing of European-adapted Andean and Chilean landraces, SD conditions are not a requirement for tuberisation, reflecting their adaptation to long day (LD) environments typical of Eurasia (Gutaker et al., 2019).

Photoperiodic regulation of tuberisation shares mechanisms closely related to the floral transition (Navarro et al., 2011). In the leaves and under SD conditions, phytochrome F (StPHYF) interacts with phytochrome B (StPHYB) to contribute to the light-dependent regulation of StCOL1 (a homolog of CONSTANS [CO]), which then activates SELF PRUNING 5G (StSP5G) in LD conditions (Zhou et al., 2019). StSP5G acts as an antagonist of SELF PRUNING 6A (StSP6A), an ortholog of *Arabidopsis* FLOWERING LOCUS T (FT) and a key mobile signal (tuberigen) for tuberisation (Abelenda et al., 2016) (**Figure 2**). Other important components of this regulatory network are the tuberisation factor StMADS1 (*Solanum tuberosum* MADS-box 1) and the CYCLING DOF FACTOR (CDF). CDFs are circadian-regulated transcription factors that modulate *StCOL1* expression and indirectly influence *StSP6A* expression, thereby linking environmental cues to developmental timing (Abelenda et al., 2016; Fornara et al., 2009; Imaizumi et al., 2005; Navarro et al., 2011).

**Figure 2:**
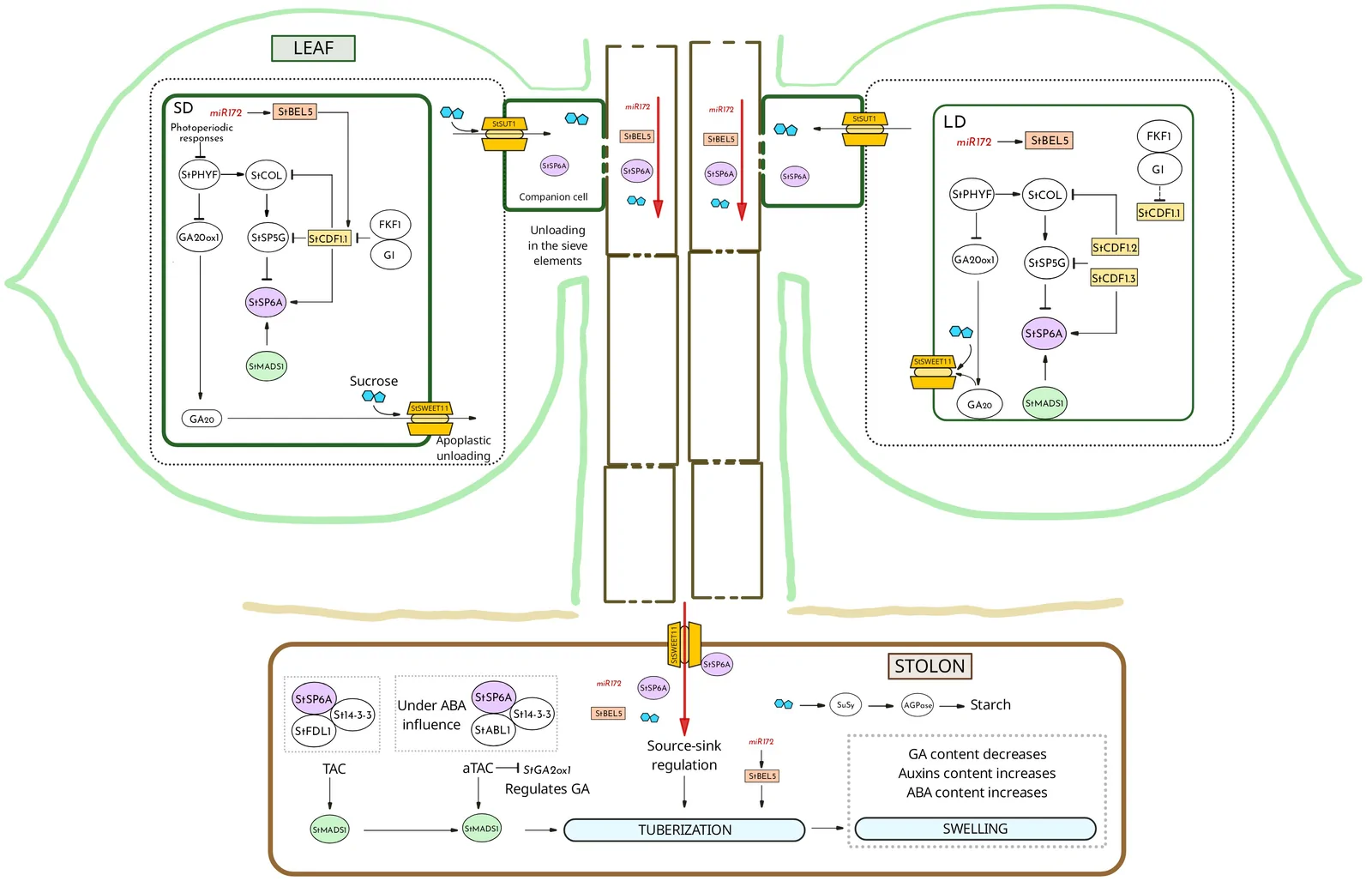
Induction of tuberisation under contrasting photoperiods, highlighting the role of *Solanum tuberosum CYCLING DOF FACTOR* (*StCDF*) alleles. Under short-day (SD) conditions, *StCDF1* promotes tuberisation by repressing CONSTANS (CO), which reduces expression of SELF-PRUNING 5G (StSP5G), a repressor of the tuberigen SELF-PRUNING 6A (StSP6A). During SD, *phytochrome F* (*StPHYF*) is not up-regulated and therefore cannot stabilise CO, further enabling StSP6A accumulation. StCDF1 is normally targeted for degradation by the FLAVIN-BINDING KELCH REPEAT F-BOX PROTEIN 1 (FKF1) and GIGANTEA (GI) complex (FKF1-GI). Under long-day (LD) conditions, however, allelic variants *StCDF2* and *StCDF3* can escape FKF1–GI mediated degradation, allowing tuberisation even in non-inductive photoperiods. After tuber induction, several mobile signals, including microRNA172 (miR172), BELLRINGER-1-like 5 (StBEL5) mRNA, StSP6A protein, and sucrose, are loaded into phloem sieve elements and transported from leaves to stolon tips. There, they activate tuber formation through the tuberigen activation complex (TAC) and the alternative TAC (aTAC), and initiate starch biosynthesis via enzymes such as sucrose synthase (SuSy) and ADP-glucose pyrophosphorylase (AGPase).

Amongst CDFs, StCDF1 is critical for photoperiod-dependent tuberisation. The ancestral *StCDF1.1* allele supports late tuberisation through interactions with the blue-light receptor FLAVIN BINDING KELCH REPEAT F-BOX PROTEIN 1 (FKF1) and the clock gene product GIGANTEA (GI), whereas the *StCDF1.2* and *StCDF1.3* alleles contain insertions that truncate the protein and disrupt interaction with FKF1, leading to increased protein stability and earlier tuber initiation (Kloosterman et al., 2013). Although *StCDF1* has not been implicated in endodormancy regulation, the effect of *StCDF1* alleles on tuber developmental timing raises the possibility that they may also influence endodormancy duration. For example, genotypes carrying the late-tuberising *StCDF1.1* allele might also exhibit longer periods of endodormancy, whereas those carrying the earlier-tuberising *StCDF1.2* or *StCDF1.3* alleles might exhibit shorter endodormancy periods. However, this hypothesis has not been tested experimentally. Consequently, the potential use of photoperiod-regulated pathways as tools for endodormancy manipulation remains speculative and warrants further investigation. Future studies should determine whether tuberisation timing and endodormancy duration are genetically linked, and whether any such relationship is consistent across genotypes and environments.

Following the tuberisation induction pathway, and in addition to StSP6A, other mobile signals are involved in regulating this process. StBEL5, a transcription factor encoding for a mobile mRNA that accumulates in stolon tips, regulates genes involved in GA catabolism (*StGA2ox1*), auxin synthesis (*YUCCA1a*), and cytokinin (CK) biosynthesis (*IPT*) and activation (*LOG*), thereby modulating growth and development (Lin et al., 2013; Sharma et al., 2016). The StBEL5 activity can be modulated by the phloem-mobile miR172, which acts as a long-distance signal. Notably, miR172 levels increase under SD conditions and specifically rise in stolons at the onset of tuber formation (Martin et al., 2009).

The mobilisation of these signals occurs primarily through the phloem. Sucrose loading mediated by transporters such as StSUT1, and StSWEET11, is required for providing carbon to developing tubers. Under inductive conditions, StSP6A binds to StSWEET11 in the stolons, preventing the passive loss of sucrose into the apoplastic space and redirecting sugar flux to the developing tuber. The direct physical interaction represents a link between photoperiod and sugar metabolism pathways in the regulation of tuber formation in potato (Abelenda et al., 2019). In stolons, StSP6A binds with FLOWERING LOCUS D-like 1 (StFDL1) to form the Tuberigen Activation Complex (TAC) (Teo et al., 2017). Alternatively, ABA promotes tuberisation by forming an alternative TAC (aTAC), in which StABL1 interacts with StSP6A to modulate GA levels through downregulation of *StGA2ox1* (Jing et al., 2022; Mathura et al., 2024). Both TAC and aTAC promote tuberisation by regulating key transcription factors involved in the initiation and development of tubers (**Figure 2**). They facilitate the redistribution of resources and the coordination of hormone signalling to promote tuber formation (Mathura et al., 2024). Recent evidence indicates that these complexes also act through a complementary, non-transcriptional mechanism via the induction of *GERMIN3*, a downstream target of TAC that enhances the movement of assimilates and signalling molecules into the developing stolon apex, thereby linking TAC/aTAC activity with the establishment of sink strength and meristem activation required for tuber initiation (Campbell et al., 2025). Overexpression of *GERMIN3* was associated with earlier tuber initiation and increased tuber yield in *Solanum andigena*, whilst similar increases in tuber yield were observed in cv. Desireé. This overexpression also increased the number of sprouts per tuber after 160 days of storage at 10 °C in both genotypes, but neither ecodormancy nor endodormancy break was evaluated in this study.

Finally, after this inductive process, the stolon begins to swell, accompanied by a reorientation of cell division from transverse to longitudinal in the subapical zone of stolon tips. This transition is regulated by a complex hormonal crosstalk, particularly between auxin and GAs (Roumeliotis et al., 2012). During swelling, auxin levels increase whilst GA content decreases, facilitating tuber initiation (Roumeliotis et al., 2012). Following this step, the tuber enters the bulking stage.

### 4.3 Metabolic shift to allocate sugars in developing tubers

Carbon metabolism plays a pivotal role in the transition from tuber initiation to bulking. This process involves the efficient distribution and utilisation of photo assimilates for energy production, biosynthesis, and starch storage (Fernie and Willmitzer, 2001; Viola et al., 2007). During this transition, the plant undergoes a metabolic shift that prioritises carbohydrate allocation to developing stolons.

Under inductive conditions, the photoassimilates synthesised in the leaves, and the long-distance signals that induce tuberisation (e.g. StSP6A, StBEL5, and miR172), travel from the leaves to the stolon’s tips, where tubers form (Banerjee et al., 2006; Martin et al., 2009; Navarro et al., 2011). Upon arrival at the stolon, a portion of the mobilised carbon is used for respiratory metabolism to generate ATP and support biosynthetic processes. Another portion is allocated to the synthesis of cell wall components, such as cellulose, hemicellulose, and pectin, which are essential for cell division and expansion during tuber growth. However, at tuber maturity, over 70% of the carbohydrates are stored as starch (Abelenda et al., 2019; Fernie and Willmitzer, 2001).

Sucrose cleavage by acid invertase generates glucose and fructose, maintaining a sucrose concentration gradient that promotes further sucrose import and provides carbon for subsequent starch synthesis (Appeldoorn et al., 1999). Glucose and fructose are directed into the amyloplasts (**Figure 3**), where starch biosynthesis begins, and then glucose (glucose-1-phosphate) is converted into ADP-glucose (adenosine diphosphate glucose), which serves as the substrate for starch biosynthesis, through the action of the enzyme ADP-glucose pyrophosphorylase (AGPase) (Ahmad et al., 2024).

**Figure 3:**
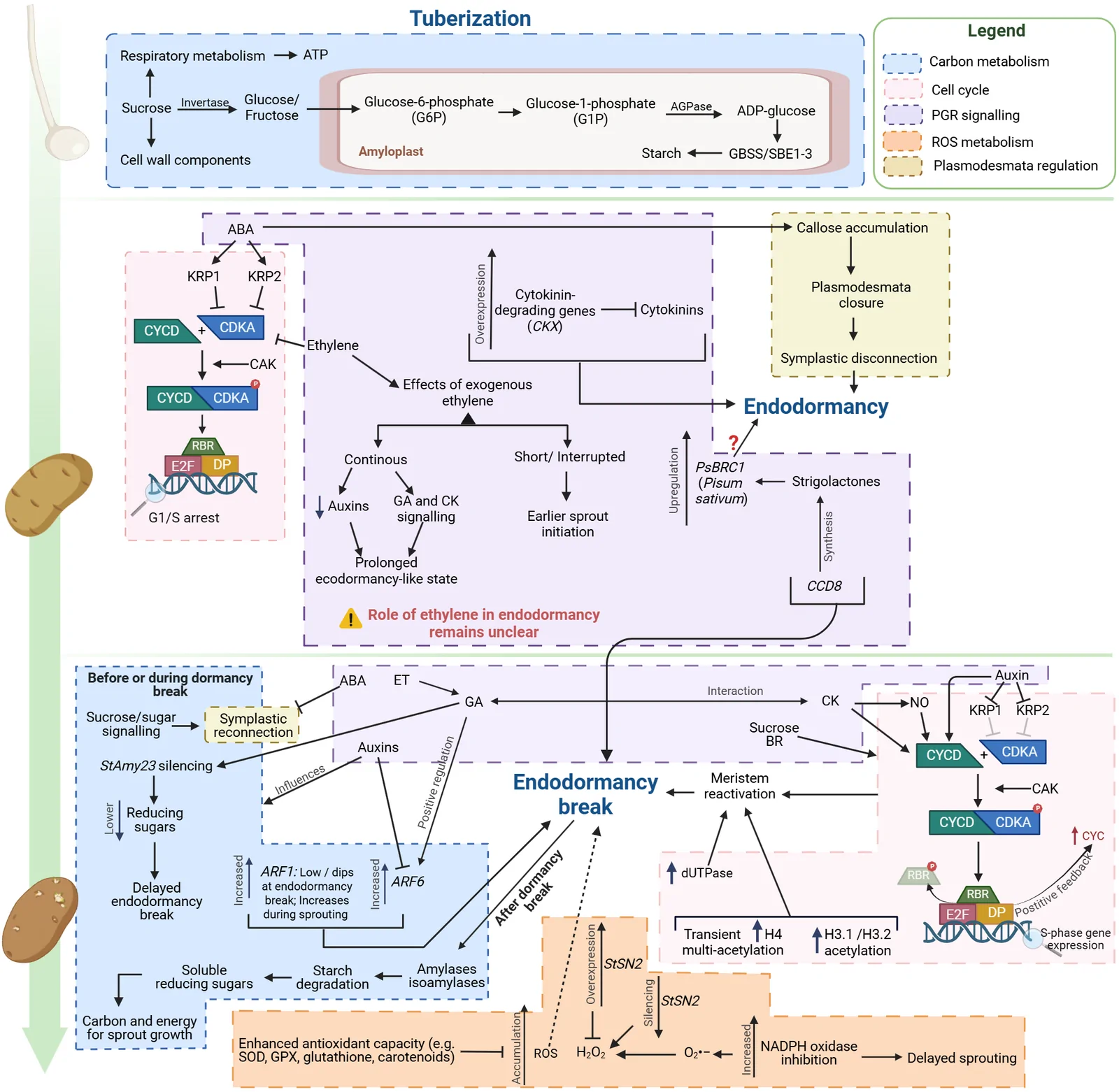
Overview of the metabolic, hormonal, cellular, and regulatory transitions occurring in potato tubers during tuberisation, endodormancy, and endodormancy break. The figure summarises the coordinated regulation of carbon metabolism and starch turnover, cell-cycle progression, plant growth regulator signalling, reactive oxygen species (ROS) homeostasis, plasmodesmata connectivity, and epigenetic modifications associated with the induction, maintenance, and release of dormancy. During tuberisation, sucrose is converted into starch, and cell proliferation is restrained by ABA-mediated regulation of the G1/S transition. Endodormancy is characterised by hormonal control involving ABA, ethylene, cytokinins, and strigolactones, together with plasmodesmata closure and symplastic disconnection. Endodormancy break is associated with starch degradation, soluble sugar accumulation, meristem reactivation, chromatin remodelling, ROS signalling, and re-entry into the cell cycle, ultimately supporting sprout growth. Arrows indicate activation, stimulation, transport, or metabolic fluxes, whereas blunt-ended lines indicate inhibition or repression. Dashed arrows represent indirect or proposed regulatory relationships. Abbreviations: G6P, glucose-6-phosphate; G1P, glucose-1-phosphate; ADP-glucose, adenosine diphosphate glucose; AGPase, ADP-glucose pyrophosphorylase; GBSS, granule-bound starch synthase; SBE, starch branching enzyme; ABA, abscisic acid; ET, ethylene; GA, gibberellin(s); CK, cytokinin(s); KRP1/2, Kip-related proteins 1 and 2; CYCD, D-type cyclins; CDKA, cyclin-dependent kinase A; CAK, CDK-activating kinase; RBR, retinoblastoma-related protein; E2F, E2F transcription factor; DP, dimerisation partner; NO, nitric oxide; BR, brassinosteroid(s); ROS, reactive oxygen species; CAT, catalase; APX, ascorbate peroxidase; GPX, glutathione peroxidase; FT, FLOWERING LOCUS T; *PsBRC1*, *BRANCHED1 transcription factor from Pisum sativum*; *ARF1* and *ARF6*, *auxin response factors 1* and *6*; *CCD8*, *carotenoid cleavage dioxygenase 8*; TrxG complex, Trithorax group protein complex; LDL1/2, LYSINE-SPECIFIC DEMETHYLASE LIKE 1 and 2; *StAmy23*, *α-amylase 23*; *StSN2, Snakin-2*; dUTPase, deoxyuridine triphosphatase.

ADP-glucose is then used by granule-bound starch synthase (GBSS) to elongate the growing starch chains. GBSS adds glucose units through α-1,4- glycosidic bonds, forming the linear polysaccharide known as amylose. Simultaneously, branching enzymes (SBE1, SBE2 and SBE3) create α-1,6-glycosidic bonds, leading to the formation of amylopectin, the branched form of starch (Ahmad et al., 2024). Total potato tuber starch typically consists of 20-30% amylose and 70-80% amylopectin, and this ratio determines the starch properties (Smith et al., 1997). The proportion of these polysaccharides depends on the genetic background and affects the quality of potato tubers. As it will be discussed in Section 6.1, endodormancy break is primarily associated with sugar signalling and symplastic reconnection, whilst starch degradation will contribute to the increased carbon and energy demands of bud growth following dormancy break.

### 4.4 Internal regulation of tuberisation, endodormancy and endodormancy break

Although tuberisation has been discussed up to this section, there are many internal factors that regulate it and the subsequent stages (endodormancy and endodormancy break). Numerous processes are involved, including but not limited to signal transduction, sucrose transport, hormone synthesis, cell division, tissue differentiation, sink-source balance, energy storage, and stolon branching (Shan et al., 2013; Zhang et al., 2024). Understanding the regulation and crosstalk between the various regulatory components that govern each process and the progression from one state to another (from tuberisation to endodormancy and subsequently to endodormancy break) is highly challenging, making it difficult to identify the precise elements that act as “key regulators”.

**Supplementary Table 1** summarizes updated information from the literature regarding the regulatory components that play a role in each stage, their main functions, and their crosstalk with other regulatory components. Whilst many of these have been mentioned and will continue to be mentioned throughout this review, the table serves as a basis for identifying regulatory components that may be considered as future targets for genetic engineering to better understand the regulation of dormancy.

## 5 Endodormancy

As tubers mature, their metabolic activity gradually decreases, marking the transition from active to inactive storage organs, a process known as endodormancy establishment. During potato tuber development, the stolon undergoes radial expansion, whilst axillary buds located at each node develop into the eyes of the tuber. It is suggested that endodormancy is influenced by bud age, with the youngest bud, formed closest to the apex of the stolon, being the last to enter endodormancy (Claassens and Vreugdenhil, 2000). As endodormancy sets in, the other buds on the tuber complete their development and may become endodormant as well.

One key aspect of endodormancy progression is the symplastic disconnection that occurs between the dormant bud (meristem) and surrounding parenchymatic tissue. This process is mediated by the control of plasmodesmata (PD), which regulates the movement of solutes and signalling molecules essential for bud activation and growth. This isolation, initially described in vegetative buds of birch (*Betula pubescens*) (Rinne et al., 2001), is primarily achieved through the deposition of callose at plasmodesmata, effectively blocking intercellular transport. A similar mechanism has been confirmed in potato (*S. tuberosum*, cv. Désirée), where dormant buds are symplastically isolated from surrounding tissues and symplastic connectivity is restored prior to dormancy break (Sonnewald and Sonnewald, 2014; Viola et al., 2007). In addition, sucrose unloading into the bud has been proposed as a prerequisite for bud growth, emphasising the importance of re-establishing symplastic transport during dormancy release (Sonnewald and Sonnewald, 2014). More recently, Danieli et al. (2023) showed that ABA and sugar accumulation act antagonistically in regulating this process. Exogenously applied ABA to detached apical buds promoted plasmodesmata closure and maintenance of endodormancy. In contrast, higher sugar accumulation favoured plasmodesmata reopening, restoration of symplastic connectivity, and bud growth. These effects were demonstrated at 14 °C, where sprouting was quantified as the time required for 50% of tubers to produce at least one bud reaching 2 mm in length.

### 5.1 G0 and G1 phase of cell cycle are key for endodormancy

Endodormancy is characterised by the arrest of cells in the G1/G0 phase of the cell cycle, indicating a state of suspended growth activity (Campbell et al., 1996). During this phase, the cells remain metabolically active, but do not progress towards cell division. The cell cycle is controlled by cyclin-dependent protein kinases (CDKs), which are enzymes that control the progression of the cell cycle by phosphorylating target proteins. CDKs must associate with regulatory subunits called cyclins to become active (Inzé and De Veylder, 2006). Cyclin–CDK activity is regulated by multiple processes, including phosphorylation of CDKs, binding of CDK inhibitors (CKIs) (Inzé and De Veylder, 2006), transcriptional control of CDK regulators, and targeted protein degradation (Velappan et al., 2017).

Campbell et al. (1996) reported that potato endodormancy could be controlled indirectly via post-transcriptional modulation of genes governing cell-cycle progression, thereby restricting cell division in dormant meristems. Dormancy status was assessed at 20 °C after 2 weeks, with tubers classified as endodormant (0% sprouting), partially dormant (approximately 50% sprouting), or non-dormant (100% sprouting). During the G1 phase, the presence of growth-promoting factors like sucrose, auxin, and CK triggers the expression of genes responsible for producing proteins involved in cell cycle progression, notably A-type cyclin-dependent kinase (CDKA) and D-type cyclins (CYCD). These proteins form a complex called CDKA/CYCD, which is essential for driving the cell cycle forward (**Figure 3**) (Velappan et al., 2017).

Activation of the CDKA/CYCD complex requires phosphorylation, which is facilitated by the CDK-activating kinases (CAK) pathway. Certain substances, like ABA, act as mitotic inhibitors. ABA can deactivate the CDKA/CYCD complex by promoting the production of proteins known as kip-related proteins (KRPs), which are part of the cyclin-dependent kinase inhibitors (CKIs). KRPs inhibit the activity of CDKA/CYCD. This inhibition leads to a halt in cell cycle progression at the G1/S checkpoint, preventing cells from entering the S phase (Velappan et al., 2017), and therefore possibly preventing endodormancy break.

Campbell et al. (2012), using DMN-treated Russet Burbank tubers stored at 3–4 °C and subsequently transferred to 20 °C until 100% of the tubers had sprouted (not defined), found that DMN reduced thymidine incorporation into DNA by approximately 50%, downregulated several genes associated with cell-cycle progression, including *CYCD3*, *CDKB1*, and *PCNA*, and induced the cyclin-dependent kinase inhibitors KRP1 and KRP2. These results suggest that DMN suppresses sprout growth by maintaining meristematic cells in a G1/S cell-cycle arrest, likely through inhibition of cyclin-CDK activity. Subsequent transcriptomic studies further supported this mechanism. Campbell et al. (2020) reported that DMN represses genes associated with DNA replication, DNA methylation, cell division, and cell-cycle progression, particularly during ecodormancy, whilst inducing stress- and defence-related pathways, including salicylic acid and jasmonic acid signalling and responses. Dobry and Campbell (2025) observed that these responses were generally stronger and more consistent during ecodormancy than endodormancy across the cvs. Coomba, LaChipper, and Lamoka, and additionally reported repression of auxin-, CKs-, BRs-, and cell-cycle-related genes. More recently, Dogramaci et al. (2026a) demonstrated that DMN responses are highly tissue-specific and involve coordinated changes in hormone signalling and meristem activity, particularly following multiple DMN applications during storage.

Additionally, two compounds affecting sprouting through the regulation of the cell cycle have been investigated. Exogenous cytokinin application (1-(α-ethylbenzyl)-3-nitroguanidine) to dormant potato tubers stored at 4 °C and subsequently transferred to 20 °C induces *CYCD3* expression within days, promoting the transition to sprouting. This upregulation facilitates the release of the G1/S cell cycle arrest characteristic of dormant meristems, thereby enabling cell division and sprout elongation (Campbell et al., 2014). In contrast, methyl jasmonate (MeJA) has been shown to suppress sprouting in Russet Burbank tubers during cold storage (8 °C), partly through the downregulation of cyclins/CDKs and the upregulation of Retinoblastoma-related (RBR) proteins, which prevent the G1/S phase transition and ultimately promote cell cycle arrest (Dogramaci et al., 2024b).

Although these studies have helped elucidate the role of the cell cycle in dormancy regulation, current evidence has largely focused on cell cycle arrest in tubers that have already initiated sprouting, and mainly in cold storage. It is therefore important to elucidate and understand cell-cycle changes during endodormancy to determine whether these changes can be detected before dormancy release. Identifying early cell-cycle markers associated with dormancy break would enable the prediction and modulation of endodormancy duration, rather than relying on visible sprout emergence for management decisions.

### 5.2 ABA dominates endodormancy maintenance, whilst strigolactones support it and ethylene plays dual effects

Abscisic acid is commonly used as an indicator of dormancy status because high ABA levels are associated with growth arrest and the maintenance of endodormancy, whereas declining ABA levels accompany endodormancy break and sprout growth (Destefano-Beltrán et al., 2006; Simko et al., 1997; Suttle, 2004). Zheng et al. (2024) compared the transcriptomic and metabolomic profiles of dormant, budding, and sprouting tubers of potato cv. Atlantic (20 °C) and identified differential expressions of genes involved in ABA and other hormone signalling pathways during the transition from endodormancy to sprouting. These findings suggest the key role for ABA signalling in the regulation of tuber dormancy and sprouting.

Because ABA is generally considered the dominant hormone maintaining dormancy, disentangling its activity from other metabolic and signalling pathways is highly challenging. As discussed throughout this review, ABA interacts with and regulates numerous physiological processes. Consequently, the discussion of ABA has been distributed across multiple sections of the review. In contrast, this section focuses on other regulators that may contribute to endodormancy maintenance, particularly strigolactones and ethylene.

Strigolactones (SL) are phytohormones, that can regulate multiple developmental processes in plants and respond to various environmental stimuli (Zwanenburg and Blanco-Ania, 2018). In pea (*Pisum sativum*), SLs and CKs have been shown to act antagonistically in axillary buds through the transcriptional regulation of *PsBRC1*, a key regulator of bud outgrowth. SLs promote bud dormancy, or the inhibition of bud outgrowth, by upregulating *PsBRC1* expression, whereas CKs stimulate bud outgrowth by repressing *PsBRC1* (Dun et al., 2012). In 2013, Pasare et al. explored the effect of SL on potato cell cycle including endodormancy and sprouting, by silencing the *CAROTENOID CLEAVAGE DIOXYGENASE8* (*CCD8*) gene, key in the SLs biosynthesis pathway, they found that *CCD8*-RNAi tubers sprouted earlier (assessed at room temperature as the percentage of sprouted tubers after 12 weeks of storage). Although the role of SLs in tuber endodormancy has started to be recognised, their precise role remains unclear and requires additional study.

Although the role of ABA is relatively well described, the role of ethylene in potato tuber endodormancy and endodormancy break remains unclear. In contrast, ethylene has been more extensively studied during ecodormancy, although important mechanistic gaps still persist. Whilst some studies have reported that ethylene is neither necessary nor sufficient to break dormancy in cv. Russet Burbank minitubers (Suttle, 2009), other research suggests that its effects on sprouting depend on exposure duration and concentration. As such, short-term exposure to low ethylene concentrations (0.02–20 ppm), particularly around 2 ppm, has been shown to promote sprout initiation and shorten the duration of dormancy (Rylski et al., 1974), whereas other research suggests that continuous ethylene exposure generally suppresses sprout growth and prolongs a dormancy-like state during storage (Dai et al., 2016; Dobry and Campbell, 2025; Dogramaci et al., 2026b, 2024; Foukaraki et al., 2016a; Tosetti et al., 2021). Importantly, this suppression may not reflect maintenance of true endodormancy but rather ecodormancy maintenance (Foukaraki et al., 2016a, 2014). Collectively, these evidence supports a dual role for ethylene, whereby transient exposure promotes sprouting, whilst prolonged exposure suppresses sprout growth, with outcomes depending on tissue-specific responses, endogenous ethylene levels, and treatment conditions (Dogramaci et al., 2026b).

At the molecular level, most available transcriptomic and metabolomic data derives from studies conducted on ecodormancy. These analyses indicate that continuous exogenous ethylene contributes to ecodormancy maintenance by repressing growth-promoting hormonal pathways (auxin, CK, and GA signalling), whilst activating networks involving ABA, jasmonic acid, and ethylene response factors (Dogramaci et al., 2026b). Tosetti et al. (2021) demonstrated that 1-MCP mitigated ethylene-induced sweetening whilst largely preserving ethylene-mediated sprout suppression, suggesting that sugar accumulation and sprout inhibition may be regulated through partially distinct pathways. In this study, sprouting was assessed at 8.5 °C using a 10% peeping threshold as the phenological trigger for treatment transitions. Nevertheless, the molecular mechanisms underlying this apparent separation of sweetening and sprout suppression remain unresolved. Conversely, short-term or discontinuous ethylene exposure has been reported to accelerate sprouting, underscoring the importance of treatment duration and concentration (Suttle, 2009). These findings indicate that ethylene does not intrinsically trigger ecodormancy break but rather prolongs it through metabolic and hormonal reprogramming. Nevertheless, as most research has focused either on ecodormancy or on sprouting, rather than directly assessing endodormancy break, the role of ethylene in the regulation and termination of endodormancy remains unclear and warrants further investigation.

## 6 Endodormancy break

Endodormancy break has been proposed as a partial reverse of tuber initiation (Claassens and Vreugdenhil, 2000). Some metabolic and biochemical processes display opposite trends: reserve accumulation during tuberisation (e.g. starch synthesis and storage) is reversed during sprouting through starch mobilisation into soluble sugars, accompanied by shifts in carbohydrate-metabolising enzyme activities. In contrast, hormonal regulation is only partly reversed; whilst GAs generally shows opposing dynamics, other hormones such as ABA, CKs and ethylene display non-symmetric patterns between tuberisation and endodormancy break. Overall, endodormancy break is regulated by an interaction between endogenous and environmental signals, coordinated by transcriptional and metabolic changes (increase in metabolic and respiratory rates), where phytohormones play a fundamental role (Saidi and Hajibarat, 2021). The following sections discuss the metabolic processes involved in bud reactivation and subsequent sprout development.

### 6.1 From tubers to sprouts: sugars signal endodormancy break

Starch accumulated during tuber development is the main carbohydrate reserve. During endodormancy, overall tuber metabolic activity is low; however, during progression to endodormancy break, sucrose becomes the dominant sugar in tuber storage parenchyma (Viola et al., 2007). Evidence suggests that sugars are not only metabolic substrates but may also function as signalling molecules involved in endodormancy break. This signalling function may contribute to the regulation of symplastic reconnection between the storage parenchyma and the bud meristem. In particular, this reconnection has been proposed as a key event preceding bud outgrowth. According to Danieli et al. (2023) there is an antagonistic relationship between sugars and ABA. The increase in sugar levels promotes symplastic reconnection, whereas high ABA levels in apical buds and/or surrounding tissue inhibit it, thereby prolonging endodormancy. Eventually, sugar signalling surpasses ABA-mediated repression, leading to the restoration of symplastic connectivity and the resumption of metabolite transport to the meristem.

In addition to the proposed signalling role of sugars, the upregulation of genes involved in sucrose biosynthesis may contribute to increased sucrose accumulation, which has been proposed to facilitate the transition from endodormancy to active growth. Recently, *StSPS1* (*Sucrose Phosphate Synthase 1*) was proposed as a regulator of potato tuber vigour and endodormancy break. Cai et al. (2023) stored tubers from seven cultivars with contrasting endodormancy periods in darkness at room temperature. Endodormancy break was defined as the stage at which approximately 10% of tubers had sprouted, thus using the onset of visible sprouting as an indicator of endodormancy break. Their findings showed that *StSPS1* expression increased during the transition from endodormancy to sprouting and was negatively correlated with endodormancy duration, whilst SPS enzyme activity also increased during sprouting. Because SPS is a key enzyme involved in sucrose biosynthesis, these results suggest that *StSPS1* may contribute to sucrose accumulation, potentially supporting sugar signalling processes associated with the restoration of symplastic connectivity preceding bud outgrowth. However, the evidence is currently based on gene-expression patterns, enzyme activity measurements, and correlations amongst cultivars, and therefore remains associative rather than demonstrably causal. Functional validation is still required to confirm the direct role of *StSPS1* in endodormancy regulation.

Alongside ABA-mediated regulation, *GERMIN3* has recently been proposed as a factor influencing plasmodesmatal gating and symplastic connectivity. This role was demonstrated through plasmodesmatal gating assays in *Nicotiana benthamiana*, whilst evidence in potato is currently based on phenotypic effects on tuber initiation, sprouting, and axillary bud activation (Campbell et al., 2025). These observations suggest that *GERMIN3* promotes meristem activation, potentially through a mechanism complementary to ABA-regulated callose deposition. Taken together, these findings highlight plasmodesmatal connectivity as a potential target for controlling sprouting. Whilst ABA, sugars, and possibly *GERMIN3* appear to influence the reopening of symplastic pathways, further validation in potato, a more detailed understanding of the underlying molecular mechanisms, and direct evidence linking plasmodesmatal regulation to delayed sprouting are required before this knowledge can be translated into practical sprouting-management strategies.

Once symplastic reconnection has been established and endodormancy has been released, a sustained supply of carbon is required to support bud growth and sprout elongation. Starch degradation therefore becomes an important metabolic process for ensuring sugar availability. This breakdown is mediated primarily by amylases and isoamylases, and several studies have linked these enzymes to sprouting in potato tubers. Treatment with GA_3_ has been shown to increase amylase activity and promote ecodormancy break and sprout growth (assessed at 8 °C when sprout length reached 2 mm), suggesting that the mobilisation of starch reserves contributes to the accumulation of soluble sugars required for growth reactivation (Rentzsch et al., 2012). Additionally, silencing the α-amylase gene *StAmy23* in cv. Solara, has been shown to delay endodormancy break (assessed at 23 °C when sprout length reached 1 mm) by one to two weeks, and resulted in reduced concentrations of reducing sugars together with increased levels of malto-oligosaccharides beneath the bud eye (Hou et al., 2019). These findings suggest that *StAmy23* contributes to starch mobilisation and sugar supply during the transition from endodormancy to active bud growth.

Whilst α-amylase hydrolyses the α-1,4 glycosidic bonds of starch polymers, isoamylases target the α-1,6 glycosidic bonds, located at branch points. In plants, three isoamylase isoforms have been identified: ISA1 and ISA2 are primarily involved in starch biosynthesis, whilst ISA3 facilitates starch degradation (Hussain et al., 2003). Using RNAi, Ferreira et al. (2017) silenced all three isoforms in potatoes cv. Solara and observed reduced starch content and granule size, accompanied by increased sucrose accumulation. These changes were associated with earlier sprouting (assessed at room temperature when sprout reached 2 mm), potentially because elevated sucrose levels increased carbohydrate availability near the buds and because smaller starch granules were more readily degraded. However, whether these carbohydrate changes directly trigger endodormancy break or sprouting remains unclear.

Indeed, Viola et al. (2007) argued that starch degradation is not directly responsible for endodormancy break, because substantial starch mobilisation occurs mainly during later stages of sprouting. Their findings support the view that sugar signalling, and symplastic reconnection are more closely associated with the regulation of endodormancy break, whereas starch degradation primarily fulfils the increasing carbon and energy demands of the developing sprout after dormancy has been finished.

Following endodormancy break, further adjustments in carbohydrate metabolism support sprout growth. The A*UXIN RESPONSE FACTOR 1* (*ARF1)* has been shown to influence carbohydrate partitioning (Liu et al., 2012). Its expression remains low during endodormancy, dips at the endodormancy break stage, and then rises sharply during sprouting (assessed at 20 °C when sprout length reached 2 mm). In the latter stage, *ARF1* likely facilitates the conversion of starch into sugars and may also contribute to the redistribution of phytohormones from storage tissues to the growing bud. Consequently, Liu et al. (2012) proposed *ARF1* as a potential marker of the end of endodormancy. However, because *ARF1* expression increases mainly during active sprout growth, its utility as an early indicator of endodormancy status may be limited. Biomarkers associated with processes that precede visible sprouting, such as ABA decline, sugar signalling, and the restoration of symplastic connectivity, may provide more informative predictors of endodormancy break. In this context, *GERMIN3* represents a promising candidate, given its proposed role in regulating plasmodesmatal gating and promoting symplastic connectivity. Nevertheless, its potential as an early dormancy marker will require further validation in potato and direct confirmation of its involvement in plasmodesmatal regulation during endodormancy break. Markers that precede endodormancy break may be more valuable for developing strategies to extend the dormancy period; therefore, identifying and validating such markers remains an important research priority.

### 6.2 Cell cycle reactivation

The transition from endodormancy to active growth involves the reactivation of meristematic tissues and the resumption of cell-cycle activity. Approximately 70% of nuclei in endodormant potato meristems are arrested in the G1/G0 phase, indicating limited cell division activity (Campbell et al., 1996). Interestingly, despite this arrest, mRNA levels of *p34^cdc2^*, a cyclin-dependent kinase (CDK) that regulates the G1/S transition and entry into mitosis, remain unchanged during endodormancy, suggesting that cell cycle reactivation may be regulated post-transcriptionally rather than at the level of gene expression (Campbell et al., 1996).

Reactivation of the cell cycle is accompanied by activation of the DNA replication machinery required to sustain renewed meristem growth. One of the essential enzymes involved in this process is deoxyuridine triphosphatase (dUTPase). Senning et al. (2010) identified dUTPase as an early molecular marker of meristem reactivation during the transition from dormancy to sprouting. By monitoring tubers from harvest through storage under both room-temperature and low-temperature (8 °C) conditions, they captured molecular changes occurring during dormancy and its breakage. Their results showed that dUTPase expression was positively correlated with sprout initiation and increased significantly approximately one week before visible sprouting (when sprout reached 2 mm) in multiple cultivars, including Solara, Nicola, and Agria. These findings suggest that dUTPase activation precedes visible bud outgrowth and may mark the onset of dormancy release at the molecular level. Expression was highest in the sprout tip, confirming its association with meristematic activity. Furthermore, in a GA-induced sprouting assay, dUTPase expression increased earlier than that of the DNA replication marker histone H4, suggesting it is an early and robust marker of meristem reactivation. Nevertheless, no functional studies have evaluated whether dUTPase is directly involved in the regulation of endodormancy break.

In addition to changes in cell-cycle and replication-associated genes, epigenetic modifications have been closely associated with sprouting. During endodormancy progression, sustained increases in multi-acetylated histone H3.1 and H3.2 were observed, along with transient increases in H4 multi-acetylation that peaked around 4–5 months postharvest in cv. Russet Burbank (Law and Suttle, 2004). These epigenetic changes coincided with sprout growth, suggesting a link between histone acetylation and meristem reactivation. Importantly, increases in histone acetylation preceded increases in both RNA and DNA synthesis as well as visible sprout growth, leading the authors to propose histone acetylation as an early epigenetic event associated with endodormancy break. However, the study did not provide functional evidence that histone acetylation directly regulates endodormancy break, and therefore the observed relationship should be considered correlative rather than a validated regulatory mechanism.

Beyond cell-cycle and epigenetic regulation, metabolic signals may also contribute to endodormancy break. Trehalose-6-phosphate (T6P) acts as a sucrose-related signalling molecule that modulates SnRK1 activity, which has been implicated in the regulation of cell-cycle progression (Guérinier et al., 2013). In potato cv. Solara, Debast et al. (2011) used transgenic tubers with altered T6P levels and monitored sprouting (assessed at room temperature as the first appearance of visible sprout growth). Tubers with elevated T6P levels exhibited delayed sprouting, whereas tubers with reduced T6P levels sprouted earlier. In addition, reduced T6P was associated with increased expression of ABA 8′-hydroxylase, a key enzyme involved in ABA catabolism, whilst T6P was shown to inhibit SnRK1 activity *in vitro*. Based on these observations, the authors proposed that elevated T6P inhibits SnRK1 signalling and contributes to the maintenance of ABA levels, whereas lower T6P permits SnRK1 activation, promotes ABA degradation, and facilitates endodormancy break. However, although the study provides strong functional evidence that T6P influences endodormancy duration and sprouting behaviour, the complete T6P–SnRK1–ABA regulatory pathway remains to be fully validated through direct genetic and biochemical studies.

### 6.3 ROS and metabolic reactivation

Reactive oxygen species (ROS), including superoxide (O_2_•^-^) and hydrogen peroxide (H_2_O_2_), are continuously produced during cellular metabolism and by NADPH oxidases, whilst their levels are controlled by antioxidant enzymes. Whilst excessive ROS accumulation can cause oxidative damage, controlled ROS production also functions as cellular signalling (Bhate and Ramasarma, 2009; Liu et al., 2017a). In potato tubers, increasing evidence indicates that ROS are associated with both endo- and ecodormancy progression and sprouting, although their precise role as primary regulators of dormancy break or as products of the metabolic reactivation preceding sprout growth remains under debate.

The dormant state has been associated with low ROS accumulation and enhanced antioxidant capacity. In transgenic tubers of the cv. BF15 overexpressing *StDREB1* (*Solanum tuberosum* Dehydration Response Element Binding 1) or *VvWRKY2* (*Vitis vinifera* WRKY2), Chiab et al. (2023) reported extended ecodormancy (assessed at 4 °C; and ecodormancy break defined when 80% of sprouts reached 3 mm). Both transgenic lines displayed longer ecodormancy periods than wild-type tubers, accompanied by reduced H_2_O_2_ and malondialdehyde (MDA) accumulation and increased Superoxide Dismutase (SOD), Glutathione Peroxidase (GPX), glutathione, and carotenoid levels, suggesting that enhanced antioxidant capacity contributes to ecodormancy maintenance. Similarly, Hu et al. (2025) stored cv. Favorita tubers at 20 °C and monitored sprout rate, bud length, and bud weight. Treatment with 0.3 M nicotinamide (NAM) delayed sprouting by approximately 12 days, maintained higher ABA levels, increased antioxidant capacity, and reduced H_2_O_2_ and O_2_•^-^ accumulation, further supporting an association between low ROS levels and prolonged dormancy.

Prior to visible sprouting, potato tubers undergo extensive metabolic reactivation that may contribute to increased ROS production during endodormancy break. In the cv. Longshu 3, Liu et al. (2015) compared dormant tubers, endodormancy-release tubers without visible buds, and sprouting tubers (buds 2–3 mm in length) stored at 20 °C and found that the transition from dormant tubers to endodormancy-release tubers was characterised by the activation of starch degradation, glycolysis, fermentation, amino acid metabolism, protein turnover, transport processes, and redox-related pathways. These changes indicate a shift from growth arrest to active metabolism before visible sprout emergence. Consistent with this transition, Liu et al. (2017a) reported in the cvs. Russet Burbank and QR01 that ROS accumulation in bud tissues coincided with increased NADPH oxidase activity and elevated expression of two genes encoding plant NADPH oxidases (*StRbohA* and *StRbohB*) during endodormancy progression towards sprouting (assessed at 20 °C when sprout length reached 3 mm). Furthermore, inhibition of NADPH oxidase activity delayed sprouting, suggesting that ROS production is closely associated with endodormancy break. Complementary genetic evidence was provided by Deng et al. (2021) who showed that silencing *StSN2* in cv. Chuanyu 10 shortened endodormancy (~75 d) and increased H_2_O_2_ accumulation, whereas overexpression prolonged endodormancy (~105 d) and reduced H_2_O_2_ levels in tubers stored at 15 °C. Together, these studies indicate that ROS accumulation accompanies the metabolic reactivation preceding sprout growth and may participate in the regulatory processes associated with endodormancy break.

Collectively, current evidence supports the involvement of ROS in the regulatory network controlling potato tuber dormancy break. Experimental manipulation of ROS production or scavenging capacity alters dormancy duration and sprouting behaviour, supporting a signalling role for ROS during the transition to active growth. However, proteomic analyses also show that changes in carbohydrate, respiratory, and energy metabolism preceding visible sprouting, indicating that ROS accumulation may partly result from the increased metabolic activity associated with dormancy break. Therefore, rather than being exclusively signalling molecules or merely metabolic by-products, ROS likely fulfil both functions, acting as products of metabolic reactivation whilst simultaneously participating in the signalling processes that promote the transition from dormancy to sprout growth.

### 6.4 Gibberellins and cytokinins synergically signal endodormancy break

The transition from endodormancy to sprouting can be viewed as a process during which the ABA-dominated dormant state is progressively replaced by a regulatory program driven by CKs, GAs, and other signalling molecules, including auxins and jasmonic acid (JA), alongside substantial metabolic reactivation.

Zheng et al. (2024) performed transcriptomic and metabolomic analyses of potato tubers at three developmental stages: endodormancy, budding, and sprouting (assessed at 20 °C when sprout length reached 2 mm). They reported extensive activation and reprogramming of multiple hormone-signalling pathways during the transition from endodormancy to sprouting, including auxin, CK, ABA, JA, BR, and ethylene signalling pathways, suggesting substantial changes in hormonal regulation as tubers approach sprouting. Supporting this idea, Wang et al. (2022), using *StTCP15*-overexpressing, *StTCP15*-silenced, and wild-type potato lines (assessed at 23 °C when sprout length reached 2 mm), found that *StTCP15* promotes endodormancy break by reducing ABA accumulation whilst increasing GA_3_ levels. This shift in hormonal balance favours GAs- and CKs-mediated sprout growth whilst progressively weakening ABA dominance.

The interaction between GAs and CKs plays a significant role in the transition from endodormancy to sprouting. Hartmann et al. (2011) suggested that GAs can promote endodormancy break, but only when sufficient endogenous CK levels are present. This may be because CKs are able to initiate meristem reactivation, whereas sustained sprout elongation requires GA activity. Accordingly, GA-induced sprouting was associated with the activation of genes involved in cell-cycle re-entry, MAPK and auxin signalling, carbohydrate metabolism, and stress responses, together with extensive changes in ethylene-related pathways (Hartmann et al., 2011; Liu et al., 2012; Zheng et al., 2024). Notably, overexpression of cytokinin-degrading genes (*CKX*) resulted in prolonged endodormancy and an impaired response to GA treatment, further supporting the view that CKs act upstream of GAs during dormancy break and sprout development (Hartmann et al., 2011).

As endodormancy progressively weakens, the bud meristem acquires the capacity to resume growth, and GAs may become major regulators of visible sprout emergence and elongation by promoting growth processes and the mobilisation of stored reserves. Evidence from tubers treated 10 days after harvest with GA_3_ (10 mg L^-^¹) and subsequently stored in darkness at 5, 10, or 20 °C showed that sprouting was significantly accelerated by GA_3_ application, particularly at 10 and 20 °C (Alexopoulos et al., 2007). The authors also observed increased respiratory activity and glucose accumulation around the buds prior to visible sprouting, suggesting that GAs promote reserve mobilisation and sink establishment in developing sprouts. Similarly, Alexopoulos et al. (2017) demonstrated that early GA_3_ application to potato plants (cv. Chacasina) promoted physiological ageing of tubers and consequently enhanced earlier sprouting (defined as the percentage of visible sprouts), supporting the concept that gibberellins favour the transition from a storage state to active growth. Particularly, sprout elongation in potato tubers is also influenced by *StCEN* expression. Morris et al. (2019) demonstrated that RNAi-mediated suppression of *StCEN* increased sprout growth, whereas *StCEN* overexpression reduced sprout elongation (cv. Desiree at 20 °C). They also found that sprouting tubers with reduced *StCEN* expression accumulated higher levels of CKs, supporting a positive association between CK accumulation and sprout growth after endodormancy break.

After dormancy has been broken and gibberellin-driven growth has been initiated, auxin signalling appears to play an important role in coordinating meristem activity and the development of the emerging sprout. Faivre-Rampant et al. (2004) demonstrated that the auxin response factor *ARF6* was not expressed in ecodormant buds (assessed at 4 °C, where dormant tubers were sampled after 2–4 weeks of storage and sprouting tubers after 8–10 weeks, with sprouting assessed by visible apical bud growth) but was strongly induced during meristem activation and sprouting. Moreover, exogenous GA_3_ enhanced *ARF6* expression and bud growth, whereas auxin (NAA) reduced both *ARF6* expression and bud development. These results suggest that *ARF6* may be positively regulated by GAs and negatively by auxin in potato. Based on these findings, the authors proposed that *ARF6* could serve as a molecular marker for meristem activation and endodormancy break. However, no recent studies have further explored the role of *ARF6* in the context of endodormancy, and the original work focused primarily on transcriptional responses rather than direct auxin quantification. Consistent with these findings, other reports have shown that tubers stored at either 20 °C or 4 °C exhibited increased expression of *ARF1*, and auxin-repressed proteins during dormancy break and sprouting (Liu et al., 2012; Zheng et al., 2024). However, these studies remain correlative and further validation is required.

Additional hormonal signalling pathways appear to reinforce and sustain the transition from dormancy to active development. The microRNA stu-miR319c has recently been identified as a positive regulator of potato tuber dormancy break through modulation of the TCP–JA signalling pathway (cv. Atlantic tubers stored at 4 °C and 22 °C). Overexpression of stu-miR319c accelerated sprouting (when sprout length reached 2 mm), whereas silencing delayed dormancy break, (defined by morphological observations and peak expression of the meristematic marker dUTPase). These effects were associated with increased JA accumulation and upregulation of JA biosynthetic genes such as *StAOC* and *StLOX4*, indicating that JA may promote the progression towards active growth (Liu et al., 2025). Similarly, Liu et al. (2012) reported that dormancy break (cv. Favorita stored at 20 °C or 4 °C) was accompanied by the induction of several hormone-responsive genes, including members of the ethylene response factor (ERF) family. ERF transcripts showed a marked increase as tubers progressed from a dormant state to visible sprout growth, indicating activation of ethylene-mediated transcriptional regulation. The upregulation of ERF, together with the identification of additional ethylene-associated transcripts, suggests that ethylene signalling is activated during dormancy break and early sprout growth. Although ethylene concentrations were not directly measured, the transcriptional activation of ERF-related pathways provides molecular evidence supporting a role for ethylene in coordinating dormancy release and subsequent sprout development.

## 7 QTLs associated with endodormancy and sprouting

Dormancy is widely recognised as a complex and polygenic trait involving numerous interacting regulatory pathways. To elucidate its genetic architecture, multiple studies have conducted QTL analyses in diverse potato populations. Several dormancy-related QTLs explaining more than 5% of the phenotypic variance have been reported (**Table 1**). However, comparisons across studies revealed that most QTLs converged within specific genomic regions rather than being distributed throughout the genome. The genetic map shown in **Figure 4** provides a visual guide including only the 32 QTLs for which either physical positions on the diploid potato DM v6.1 reference genome (Pham et al., 2020) or centimorgan (cM) positions could be projected onto this map using proportional scaling along each chromosome. Chromosomes 2, 5 and 7 emerge as the most consistently implicated regions, having been detected across independent populations, environments and experimental designs, whilst chromosomes 3 and 4 provide additional recurrent evidence.

**Table 1:** Summary of quantitative trait loci (QTLs) associated with potato tuber dormancy and sprouting traits across multiple studies.

Name	Possible position		Phen. variation expl. (%)	Allelic effects	Background of the population/QTL	Dorm. assessment	Postharv temp storage	Cite
	Location (bp)	Centimorgan (cM)						
PGSC21436_160 (Pleiotropic effect with GA3 content)	chr01	38.73	6.6 to 9.4	-6.73 and -6.31	*S. chacoense* x *S. phureja*	Sprout = 2 mm	20 °C	Naz et al. (2018)
TG234 (Epistasis with TG255)	chr02:40039994.40040495	11.3	20		Backcross (P1) *S. tuberosum* x (P2)[*S. tuberosum* x *S. berthaultii*])	Sprout = 3 mm	3 °C	Simko et al. (1997)
TG20b	chr02:36262783.36263285	~10.4	9	24	Backcross *S. tuberosum* L.	Sprout = 3 mm	13 °C	Van Den Berg et al. (1996)
TG234	chr02:40039994.40040495	11.3	31	-29	Backcross *S. berthaultii*	Sprout = 3 mm	13 °C	Van Den Berg et al. (1996)
chr02_27.1_c2_45308	chr02:25888777.25887877	16.6	10	0.7	P1 (84SD22 [*S. tuberosum* x *S. chacoense*]) X P2 ([84SD22 X *S. phureja*] x *S. berthaultii*)	Dormancy break defined as 50% of tubers with ≥2 mm sprouts	22 ± 2 °C	Bisognin et al. (2018)
GP205	chr02:5521817.5525293	45.09	6.9 to 7.5		P1 (37.6% *S. tuberosum*, 28.1% *S. chacoense*, 12.5% *S. verrucosum*, 12.5% *S. microdontum*, 6.2% *S. gourlayi*, and 3.1% *S. yungasense*.) X P2 (81.3% *S. tuberosum*, 15.6% *S. chacoense*, and 3.1% *S. yungasense*)	Score based on length of sprouts, 1 = 0.1–5 mm; 2 = 6–50 mm; 3 = 51 mm-100 mm; 4 = 101 mm-150 mm, and 5 = sprouts > 151 mm	7-9 °C	Śliwka et al. (2008)
GP321a	chr02:28072639.28073521	~34-48	7.1 to 9.2		P1 (37.6% *S. tuberosum*, 28.1% *S. chacoense*, 12.5% *S. verrucosum*, 12.5% *S. microdontum*, 6.2% *S. gourlayi*, and 3.1% *S. yungasense*.) X P2 (81.3% *S. tuberosum*, 15.6% *S. chacoense*, and 3.1% *S. yungasense*)	Score based on length of sprouts, 1 = 0.1–5 mm; 2 = 6–50 mm; 3 = 51 mm-100 mm; 4 = 101 mm-150 mm, and 5 = sprouts > 151 mm	7-9 °C	Śliwka et al. (2008)
GP26	chr02:31040863.31040923	~34-48	6.9 to 9.1		P1 (37.6% *S. tuberosum*, 28.1% *S. chacoense*, 12.5% *S. verrucosum*, 12.5% *S. microdontum*, 6.2% *S. gourlayi*, and 3.1% *S. yungasense*.) X P2 (81.3% *S. tuberosum*, 15.6% *S. chacoense*, and 3.1% *S. yungasense*)	Score based on length of sprouts, 1 = 0.1–5 mm; 2 = 6–50 mm; 3 = 51 mm-100 mm; 4 = 101 mm-150 mm, and 5 = sprouts > 151 mm	7-9 °C	Śliwka et al. (2008)
GP86	chr02:26620976.26621972	~34-48	7.0 to 7.7		P1 (37.6% *S. tuberosum*, 28.1% *S. chacoense*, 12.5% *S. verrucosum*, 12.5% *S. microdontum*, 6.2% *S. gourlayi*, and 3.1% *S. yungasense*.) X P2 (81.3% *S. tuberosum*, 15.6% *S. chacoense*, and 3.1% *S. yungasense*)	Score based on length of sprouts, 1 = 0.1–5 mm; 2 = 6–50 mm; 3 = 51 mm-100 mm; 4 = 101 mm-150 mm, and 5 = sprouts > 151 mm	7-9 °C	Śliwka et al. (2008)
Prx-3	chr02		5.2		P1 (84SD22 [*S. tuberosum* x *S. chacoense*]) X P2 (*S. phureja*)	Sprout = 2 mm	10 °C	Freyre et al. (1994)
G03.2	chr02		5.4		P1 (84SD22 [*S. tuberosum* x *S. chacoense*]) X P2 (*S. phureja*)	Sprout = 2 mm	10 °C	Freyre et al. (1994)
TG276 (Epistasis with TG65)	chr02:35641615.35642127	54.7	10.90		Backcross (P1) *S. tuberosum* x (P2)[*S. tuberosum* x *S. berthaultii*])	Sprout = 3 mm	3 °C	Simko et al. (1997)
TG66	chr03:3774921.3775424	46.48	7	-14	Backcross *S. berthaultii*	Sprout = 3 mm	13 °C	Van Den Berg et al. (1996)
TG130	chr03:1755716.1756224	43	5	19	Backcross *S. tuberosum* L.	Sprout = 3 mm	13 °C	Van Den Berg et al. (1996)
chr03_53.4_c1_16513	chr03:52006738.52008953	40.3	10.3	-10.3	P1 (84SD22 [*S. tuberosum* x *S. chacoense*]) X P2 ([84SD22 X *S. phureja*] x *S. berthaultii*)	Dormancy break defined as 50% of tubers with ≥2 mm sprouts	22 ± 2 °C	Bisognin et al. (2018)
DorE6.17	chr03	69.6	10.3	-4.2	P1 (genetic background from *S. tuberosum, S. phureja, S. vernei* etc) x P2 (*S. berthaultii*)	Sprouting defined as 80% of tubers with ≥1 mm sprouts	20 ± 1 °C.	Li et al. (2018)
DorB3.17	chr03	89.4	8	-4.8	P1 (genetic background from *S. tuberosum, S. phureja, S. vernei* etc) x P2 (*S. berthaultii*)	Sprouting defined as 80% of tubers with ≥1 mm sprouts	20 ± 1 °C.	Li et al. (2018)
TG443	chr04:63819291.63819807	62.37	8	-15	Backcross *S. berthaultii*	Sprout = 3 mm	13 °C	Van Den Berg et al. (1996)
TG123 (B allele increases ABA content, linked to longer dormancy)	chr04:2977178.2977629	23.5	4.8		Backcross (P1) *S. tuberosum* x (P2)[*S. tuberosum* x *S. berthaultii*])	Sprout = 3 mm	3 °C	Simko et al. (1997)
DorE4.6	chr04	24.3	14.3	-12.2	P1 (genetic background from *S. tuberosum, S. phureja, S. vernei* etc) x P2 (*S. berthaultii*)	Sprouting defined as 80% of tubers with ≥1 mm sprouts	20 ± 1 °C.	Li et al. (2018)
Pgm-2	chr04		5.5		P1 (84SD22 [*S. tuberosum* x *S. chacoense*]) X P2 (*S. phureja*)	Sprout = 2 mm	10 °C	Freyre et al. (1994)
TG379	chr05:1545012.1549255	19.2	7	22	Backcross *S. tuberosum* L.	Sprout = 3 mm	13 °C	Van Den Berg et al. (1996)
GP78	chr05:50108761.50109001	39.53	5.4 to 6.3		P1 (37.6% *S. tuberosum*, 28.1% *S. chacoense*, 12.5% *S. verrucosum*, 12.5% *S. microdontum*, 6.2% *S. gourlayi*, and 3.1% *S. yungasense*.) X P2 (81.3% *S. tuberosum*, 15.6% *S. chacoense*, and 3.1% *S. yungasense*)	Score based on length of sprouts, 1 = 0.1–5 mm; 2 = 6–50 mm; 3 = 51 mm-100 mm; 4 = 101 mm-150 mm, and 5 = sprouts > 151 mm	7-9 °C	Śliwka et al. (2008)
chr05_5.9_c2_47663	chr05:6069160.6069434	20.1	15.3	-20	P1 (84SD22 [*S. tuberosum* x *S. chacoense*]) X P2 ([84SD22 X *S. phureja*] x *S. berthaultii*)	Dormancy break defined as 50% of tubers with ≥2 mm sprouts	22 ± 2 °C	Bisognin et al. (2018)
DorB5.3	chr05	25.5	13.9	-12.4	P1 (genetic background from *S. tuberosum, S. phureja, S. vernei* etc) x P2 (*S. berthaultii*)	Sprouting defined as 80% of tubers with ≥1 mm sprouts	20 ± 1 °C.	Li et al. (2018)
chr05_17.5_c2_51194	chr05:17476263.17474740	33.6	16.3	-12.3	P1 (84SD22 [*S. tuberosum* x *S. chacoense*]) X P2 ([84SD22 X *S. phureja*] x *S. berthaultii*)	Dormancy break defined as 50% of tubers with ≥2 mm sprouts	22 ± 2 °C	Bisognin et al. (2018)
chr05_48.9_c2_54370	chr05:52038248.52039208	50.3	9.5	-24.5	P1 (84SD22 [*S. tuberosum* x *S. chacoense*]) X P2 ([84SD22 X *S. phureja*] x *S. berthaultii*)	Dormancy break defined as 50% of tubers with ≥2 mm sprouts	22 ± 2 °C	Bisognin et al. (2018)
6-Pgdh-3	chr05		6.9		P1 (84SD22 [*S. tuberosum* x *S. chacoense*]) X P2 (*S. phureja*)	Sprout = 2 mm	10 °C	Freyre et al. (1994)
DorB6.3	chr06	29.6	12	8.2	P1 (genetic background from *S. tuberosum, S. phureja, S. vernei* etc) x P2 (*S. berthaultii*)	Sprouting defined as 80% of tubers with ≥1 mm sprouts	20 ± 1 °C.	Li et al. (2018)
DorE6.19	chr06	77.2	8.9	-4.5	P1 (genetic background from *S. tuberosum, S. phureja, S. vernei* etc) x P2 (*S. berthaultii*)	Sprouting defined as 80% of tubers with ≥1 mm sprouts	20 ± 1 °C.	Li et al. (2018)
TG499 (B allele decreases ABA, associated with shorter dormancy)	chr07:67995522.67995968	9.49	5.10		Backcross (P1) *S. tuberosum* x (P2)[*S. tuberosum* x *S. berthaultii*])	Sprout = 3 mm	3 °C	Simko et al. (1997)
chr07_47.4_c1_79731	chr07	32.2	16	-7.9	P1 (84SD22 [*S. tuberosum* x *S. chacoense*]) X P2 ([84SD22 X *S. phureja*] x *S. berthaultii*)	Dormancy break defined as 50% of tubers with ≥2 mm sprouts	22 ± 2 °C	Bisognin et al. (2018)
A15.2	chr07:6372350.6411463		14.4		P1 (84SD22 [*S. tuberosum* x *S. chacoense*]) X P2 (*S. phureja*)	Sprout = 2 mm	10 °C	Freyre et al. (1994)
Got-2	chr07		20.4		P1 (84SD22 [*S. tuberosum* x *S. chacoense*]) X P2 (*S. phureja*)	Sprout = 2 mm	10 °C	Freyre et al. (1994)
A01.3	chr07		14.6		P1 (84SD22 [*S. tuberosum* x *S. chacoense*]) X P2 (*S. phureja*)	Sprout = 2 mm	10 °C	Freyre et al. (1994)
Ill.2	chr07		15.5		P1 (84SD22 [*S. tuberosum* x *S. chacoense*]) X P2 (*S. phureja*)	Sprout = 2 mm	10 °C	Freyre et al. (1994)
A08.2	chr07		13.4		P1 (84SD22 [*S. tuberosum* x *S. chacoense*]) X P2 (*S. phureja*)	Sprout = 2 mm	10 °C	Freyre et al. (1994)
G17.2	chr07		15.7		P1 (84SD22 [*S. tuberosum* x *S. chacoense*]) X P2 (*S. phureja*)	Sprout = 2 mm	10 °C	Freyre et al. (1994)
A01.2	chr07		15		P1 (84SD22 [*S. tuberosum* x *S. chacoense*]) X P2 (*S. phureja*)	Sprout = 2 mm	10 °C	Freyre et al. (1994)
A04.1	chr07		12.4		P1 (84SD22 [*S. tuberosum* x *S. chacoense*]) X P2 (*S. phureja*)	Sprout = 2 mm	10 °C	Freyre et al. (1994)
I17.1	chr07		13.7		P1 (84SD22 [*S. tuberosum* x *S. chacoense*]) X P2 (*S. phureja*)	Sprout = 2 mm	10 °C	Freyre et al. (1994)
TG41	chr08:55737311.55737887	81.78	10	-17	Backcross *S. berthaultii*	Sprout = 3 mm	13 °C	Van Den Berg et al. (1996)
TG41	chr08:55737311.55737887	81.78	6–10%	22 and 23	Backcross *S. tuberosum* L.	Sprout = 3 mm	13 °C	Van Den Berg et al. (1996)
Got-1	chr08		9		P1 (84SD22 [*S. tuberosum* x *S. chacoense*]) X P2 (*S. phureja*)	Sprout = 2 mm	10 °C	Freyre et al. (1994)
Est-1	chr08		5.8		P1 (84SD22 [*S. tuberosum* x *S. chacoense*]) X P2 (*S. phureja*)	Sprout = 2 mm	10 °C	Freyre et al. (1994)
GP173b	chr09	40.95	5.4		P1 (37.6% *S. tuberosum*, 28.1% *S. chacoense*, 12.5% *S. verrucosum*, 12.5% *S. microdontum*, 6.2% *S. gourlayi*, and 3.1% *S. yungasense*.) X P2 (81.3% *S. tuberosum*, 15.6% *S. chacoense*, and 3.1% *S. yungasense*)	Score based on length of sprouts, 1 = 0.1–5 mm; 2 = 6–50 mm; 3 = 51 mm-100 mm; 4 = 101 mm-150 mm, and 5 = sprouts > 151 mm	7-9 °C	Śliwka et al. (2008)
chr09_52.5_c2_42964	chr09:59234232.59236339	32.1	9.8	-24.5	P1 (84SD22 [*S. tuberosum* x *S. chacoense*]) X P2 ([84SD22 X *S. phureja*] x *S. berthaultii*)	Dormancy break defined as 50% of tubers with ≥2 mm sprouts	22 ± 2 °C	Bisognin et al. (2018)
chr11_10.8_c2_33913	chr11:11457935.11458775	38.7	12.1	-7.4	P1 (84SD22 [*S. tuberosum* x *S. chacoense*]) X P2 ([84SD22 X *S. phureja*] x *S. berthaultii*)	Dormancy break defined as 50% of tubers with ≥2 mm sprouts	22 ± 2 °C	Bisognin et al. (2018)

**Figure 4:**
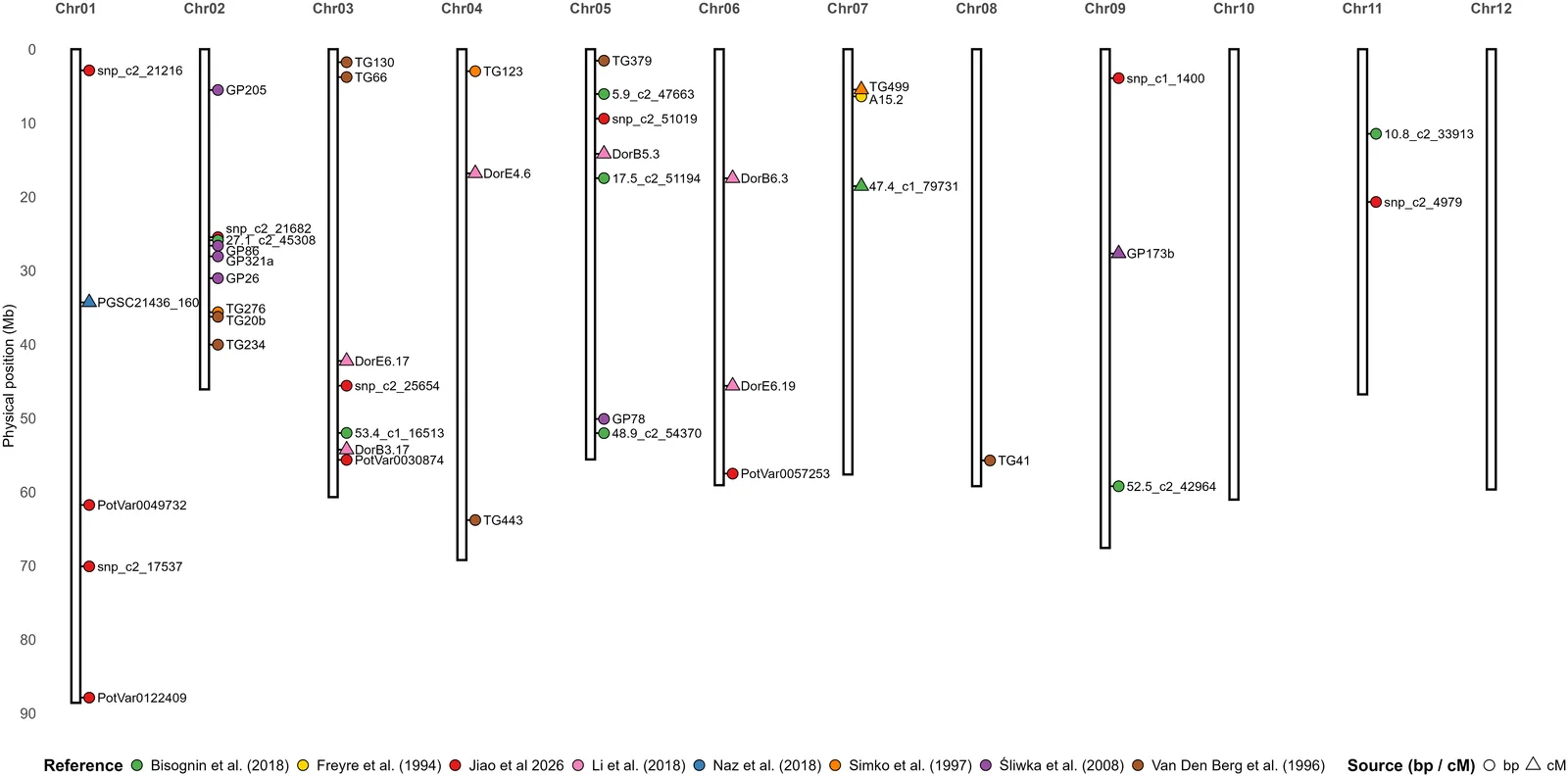
Distribution of some dormancy-related quantitative trait loci (QTLs) across the 12 potato chromosomes, mapped onto the DM v6.1 reference genome. Each circle indicates the physical (bp-based) position of QTLs reported in previous studies, colour-coded by reference (Bisognin et al., 2018; Freyre et al., 1994; Li et al., 2018; Naz et al., 2018; Simko et al., 1997; Śliwka et al., 2008; Van Den Berg et al., 1996). Triangles indicate QTLs originally reported in centiMorgans (cM), which were projected onto the physical map assuming proportional scaling along each chromosome. Some names have been shortened for ease of visualisation.

Amongst these regions, chromosome 2 represents the strongest consensus dormancy and sprouting loci currently described. QTLs in this chromosome were identified in populations derived from different *Solanum* species and were repeatedly detected by Freyre et al. (1994); Van Den Berg et al. (1996); Śliwka et al. (2008); Bisognin et al. (2018), and in tetraploid genotypes by Jiao et al. (2026). Moreover, Simko et al. (1997) mapped ABA-associated QTLs to the same chromosomal regions previously associated with potato tuber ecodormancy, providing physiological support for the involvement of ABA in sprouting regulation. In this study, one of the most important loci was the QTL linked to the marker *TG234* on chromosome 2. Although this locus was not detected as a main effect, it explained approximately 20% of the phenotypic variation in ABA content through its epistatic interaction with a QTL located on chromosome 4 near the markers *TG255*/*TG65*. Notably, tubers had been stored at 3 °C for approximately 120 days before ABA quantification, suggesting that this QTL may be particularly relevant during postharvest cold storage. The authors further reported that the allelic combinations at *TG234* and *TG255*/*TG65* showed a strong correlation between high ABA accumulation and extended ecodormancy duration, providing genetic evidence for a causal relationship between ABA signalling and tuber ecodormancy. This same epistatic interaction had previously been identified for endodormancy (13 °C) in the same mapping population, where the presence of the *S. berthaultii* allele at *TG234*/*TG276* together with the appropriate allele at *TG255*/*TG65* was associated with both increased ABA levels and longer endodormancy periods (Van Den Berg et al., 1996). These results indicate that the *TG234* region on chromosome 2 likely contributes to the control of dormancy by modulating ABA-related pathways in concert with chromosome 4 loci.

Chromosome 7 represents another important genomic region associated with endodormancy and sprouting. Freyre et al. (1994) identified multiple endodormancy-related QTLs on chromosome 7 (**Table 1**), including the largest-effect dormancy QTL, which explained up to 20.4% of the phenotypic variation (*Got-2*). Subsequent studies have consistently detected dormancy- and sprouting-related QTLs in the same genomic region. Several candidate genes involved in hormone metabolism and signalling co-localise with these QTL intervals (Bisognin et al., 2018). Amongst them, *GAD2* (2*-Oxoglutarate-dependent dioxygenase*; PGSC0003DMG400011751) and *GA2ox2* (*Gibberellin 2-oxidase 2*; PGSC0003DMG400033046) are associated with GAs metabolism. The region also contains several auxin-related genes, including *ARG7* (*Indole-3-acetic acid-induced protein*; PGSC0003DMG400013575), *GH3.6* (PGSC0003DMG400026186), and *GH3.5* (PGSC0003DMG401018368), which are involved in auxin homeostasis through IAA conjugation.

Chromosome 5 has repeatedly been associated with tuber endodormancy in different mapping populations. Li et al. (2018) identified the QTL *DorB5.3*, which was detected consistently across seven environments, suggesting a relatively stable effect. Additional analyses indicated that *DorB5.3* overlaps with a QTL associated with reducing sugars accumulation, raising the possibility that the same genomic region may influence both endodormancy duration and tuber sugar content (Li et al., 2022). Although the underlying genetic relationship remains to be fully resolved, this region could be of interest for breeding programmes because it may contribute to variation in two traits that are important for potato storage and processing quality.

Another region repeatedly associated with dormancy-related traits is chromosome 3. QTLs identified by Bisognin et al. (2018) overlap with loci subsequently detected in tetraploid germplasm by Jiao et al. (2026), suggesting that chromosome 3 contributes to the genetic control of postharvest tuber behaviour. A notable candidate gene within this region is *CDC20* (*Cell Division Cycle 20*) near the QTL peak marker snp_c2_25654 (Jiao et al., 2026). As a key regulator of cell-cycle progression and cyclin degradation, *CDC20* is a biologically plausible candidate because tuber dormancy is characterised by meristematic cell cycle arrest, which must be reactivated during dormancy break.

Despite the considerable progress made in identifying dormancy-associated QTLs, several challenges still limit the integration and practical application of these results. One of the most important is the lack of standardisation in dormancy phenotyping which is exemplified by Sharma et al. (2021). Although highly relevant to postharvest tuber biology, this study was not included in the comparative dormancy-QTL synthesis presented here because its primary focus was tuber sprout elongation and sprout-growth dynamics during storage rather than dormancy duration itself. The authors measured sprout growth at multiple postharvest time points and modelled dormancy release indirectly through sprout-development parameters. Nevertheless, the study identified QTLs on chromosomes 2, 3, 4, 5 and 10, several of which overlap with genomic regions previously associated with dormancy. In addition, candidate genes linked to sprout growth regulation, including a *CENTRORADIALIS* (*StCEN*) homologue, were identified, highlighting the complex genetic architecture underlying postharvest tuber behaviour. These findings provide valuable complementary information but also illustrate the importance of distinguishing between dormancy regulation and subsequent sprout growth when comparing genetic studies.

Beyond phenotyping differences, comparisons amongst studies are further complicated by variation in environmental conditions, storage protocols and genetic backgrounds. Most dormancy studies have been conducted in biparental diploid populations developed from distinct combinations of cultivated and wild *Solanum* species. As a result, it is often unclear whether QTLs detected in only a single study represent population-specific effects or loci controlling dormancy across a broader range of germplasms. This issue is compounded by the fact that majority of current evidence originates from diploid mapping populations. Although these populations are highly suitable for genetic analyses because of their simpler inheritance patterns, the direct application of diploid QTL discoveries to cultivated tetraploid potato is not straightforward due to allele dosage effects, more complex segregation patterns and potentially different epistatic interactions. Consequently, independent validation across populations, environments and tetraploid breeding germplasm is essential before these loci can be considered reliable targets for marker-assisted selection.

## 8 Future recommendations

One of the major challenges identified throughout this review is the lack of standardised criteria for distinguishing time of entering dormancy, dormancy maintenance, dormancy break, and sprouting amongst others. To facilitate comparisons amongst studies, or even between storage conditions, future research would benefit from a more consistent phenotyping framework. We propose that developmental stages should be clearly separated during phenotyping. Building upon the classification systems proposed by Morris et al. (2019) and Dogramaci et al. (2026b), tubers could be categorised as: (i) dormant, with no visible bud outgrowth; (ii) pre-eye movement, characterised by initial meristem activation without visible bud emergence; (iii) dormancy break, defined by visible eye movement and bud growth approaching or below 1 mm; and (iv) sprouting, characterised by active bud elongation exceeding 1–2 mm. The widespread adoption of such a developmental framework would allow dormancy maintenance, dormancy break, and sprout growth to be studied as distinct biological processes, thereby improving the interpretation and comparability of physiological, molecular, and genetic studies.

Moreover, the adoption of standardised phenotyping criteria is a prerequisite for the identification and validation of reliable biomarkers associated with specific stages of dormancy progression. A major priority for future research is the identification of phenotypic and molecular indicators capable of predicting endodormancy break before visible sprout emergence. Greater emphasis should be placed on markers associated with processes involved in dormancy maintenance and break, including ABA catabolism, sugar signalling, and the restoration of symplastic connectivity, rather than on markers that primarily reflect subsequent sprout growth. Candidate biomarkers such as dUTPase, GERMIN3, components of the T6P-SnRK1 pathway, and transcriptomic signatures associated with meristem reactivation represent promising targets. However, their predictive value remains largely unvalidated across genotypes, environments, and storage conditions. In parallel, several QTL and transcriptomic studies have identified candidate genes potentially involved in dormancy regulation. Functional validation of these candidates remains limited, although studies of genes such as *TERMINAL FLOWER1/CENTRORADIALIS* have demonstrated the value of genetic transformation approaches in establishing causal relationships with tuber sprouting. The increasing accessibility of CRISPR-based genome editing provides new opportunities to systematically validate candidate genes and regulatory networks, either individually or in combination, thereby improving our understanding of the mechanisms controlling dormancy maintenance and break.

Future efforts should also focus on establishing causal relationships amongst candidate regulators through integrated genetic, physiological, and multi-omics approaches combined with rigorous functional validation. Attention should be given to G × E interactions and the influence of preharvest environmental conditions on dormancy development. Multi-environment trials, comparative transcriptomic meta-analyses, and the integration of transcriptomic, proteomic, epigenetic, and small-RNA datasets may prove especially valuable for identifying conserved regulatory networks governing dormancy regulation. Ultimately, translating these advances into breeding applications through candidate-gene validation, marker-assisted selection, and genomic selection will be critical for developing cultivars with improved dormancy and storage performance.

## 9 Conclusions

Potato tuber endodormancy and sprouting are complex traits that are largely under genetic control. Nevertheless, preharvest environmental conditions also contribute substantially to endodormancy duration and sprouting behaviour, although their effects and underlying mechanisms remain poorly understood. Current evidence suggests that many of the factors determining endodormancy duration are established during tuber development, when long-distance signals from the aerial organs induce tuberisation and redirect carbon allocation towards the developing tuber. The establishment of endodormancy appears to be associated with symplastic disconnection and cell cycle arrest, whereas endodormancy maintenance is primarily regulated by ABA, together with interactions involving other hormonal pathways.

Endodormancy break is not a single event but rather a progressive transition involving multiple interconnected physiological and molecular processes. Available evidence suggests that changes in sugar metabolism, restoration of symplastic connectivity, and reactivation of meristematic activity occur prior to visible sprouting. Cell cycle re-entry is subsequently accompanied by a shift from ABA-dominated regulation towards the increased influence of GAs, CKs, and other growth-promoting signals. Reactive oxygen species are also consistently associated with dormancy progression, likely reflecting the re-establishment of metabolic activity, although their precise signalling role remains unresolved.

Despite significant advances in our understanding of potato tuber dormancy, important questions remain regarding the temporal sequence and causal relationships amongst symplastic reconnection, sugar signalling, hormonal regulation, ROS dynamics, and cell cycle reactivation. Collectively, current evidence suggests that potato tuber dormancy is not controlled by a single pathway but rather emerges from the interaction of multiple interconnected physiological, metabolic, and molecular processes that regulate endodormancy maintenance and break. The major challenge is therefore no longer to identify additional dormancy-associated loci, genes, hormones, or metabolites, but to understand how these factors interact within dynamic regulatory networks and how their effects are modulated by environmental conditions. Bridging these knowledge gaps will be essential to distinguish endodormancy release from visible sprouting and to improve phenotyping, storage management, and breeding strategies aimed at achieving more predictable dormancy performance.

## References

[B1] Abelenda J. A. Bergonzi S. Oortwijn M. Sonnewald S. Du M. Visser R. G. F. . (2019). Source-sink regulation is mediated by interaction of an FT homolog with a SWEET protein in potato. Curr. Biol. 29, 1178–1186.e6. doi: 10.1016/j.cub.2019.02.018 30905604

[B2] Abelenda J. A. Cruz-Oró E. Franco-Zorrilla J. M. Prat S. (2016). Potato StCONSTANS-like1 suppresses storage organ formation by directly activating the FT-like StSP5G repressor. Curr. Biol. 26, 872–881. doi: 10.1016/j.cub.2016.01.066 26972319

[B3] Ahmad D. Ying Y. Bao J. (2024). Understanding starch biosynthesis in potatoes for metabolic engineering to improve starch quality: A detailed review. Carbohydr. Polym. 346, 1–16. doi: 10.1016/j.carbpol.2024.122592 39245484

[B4] Aksenova N. P. Konstantinova T. N. Golyanovskaya S. A. Sergeeva L. I. Romanov G. A. (2012). Hormonal regulation of tuber formation in potato plants. Russ. J. Plant Physiol. 59, 451–466. doi: 10.1134/S1021443712040024

[B5] Alamar M. C. Tosetti R. Landahl S. Bermejo A. Terry L. A. (2017). Assuring potato tuber quality during storage: A future perspective. Front. Plant Sci. 8, 1–6. doi: 10.3389/fpls.2017.02034 29234341 PMC5712419

[B6] Alboresi A. Gestin C. Leydecker M. T. Bedu M. Meyer C. Truong H. N. (2005). Nitrate, a signal relieving seed dormancy in Arabidopsis. Plant Cell Environ. 28, 500–512. doi: 10.1111/j.1365-3040.2005.01292.x 16229082

[B7] Alexopoulos A. A. Akoumianakis K. A. Vemmos S. N. Passam H. C. (2007). The effect of postharvest application of gibberellic acid and benzyl adenine on the duration of dormancy of potatoes produced by plants grown from TPS. Postharvest Biol. Technol. 46, 54–62. doi: 10.1016/j.postharvbio.2007.03.01642574925

[B8] Alexopoulos A. A. Karapanos I. C. Akoumianakis K. A. Passam H. C. (2017). Effect of gibberellic acid on the growth rate and physiological age of tubers cultivated from true potato seed. J. Plant Growth Regul. 36, 1–10. doi: 10.1007/s00344-016-9616-z30311153

[B9] Appeldoorn N. J. G. De Bruijn S. M. Koot-Gronsveld E. A. M. Visser R. G. F. Vreugdenhil D. Van Der Plas L. H. W. (1999). Developmental changes in enzymes involved in the conversion of hexose phosphate and its subsequent metabolites during early tuberization of potato. Plant Cell Environ. 22, 1085–1096. doi: 10.1046/j.1365-3040.1999.00473.x37945311

[B10] Banerjee A. K. Chatterjee M. Yu Y. Suh S. G. Miller W. A. Hannapel D. J. (2006). Dynamics of a mobile RNA of potato involved in a long-distance signaling pathway. Plant Cell 18, 3443–3457. doi: 10.1105/tpc.106.042473 17189340 PMC1785412

[B11] Bentsink L. Jowett J. Hanhart C. J. Koornneef M. (2006). Cloning of DOG1, a quantitative trait locus controlling seed dormancy in Arabidopsis. PNAS 103, 17042–17047. doi: 10.1073/pnas.0607877103 17065317 PMC1636575

[B12] Bhate R. H. Ramasarma T. (2009). Evidence for H2O2 as the product of reduction of oxygen by alternative oxidase in mitochondria from potato tubers. Arch. Biochem. Biophys. 486, 165–169. doi: 10.1016/j.abb.2009.04.003 19375411

[B13] Bisognin D. A. Manrique-Carpintero N. C. Douches D. S. (2018). QTL analysis of tuber dormancy and sprouting in potato. Am. J. Potato Res. 95, 374–382. doi: 10.1007/s12230-018-9638-030311153

[B14] Bou-Torrent J. Martínez-García J. F. García-Martínez J. L. Prat S. (2011). Gibberellin a 1 metabolism contributes to the control of photoperiod-mediated tuberization in potato. PloS One 6, 1–10. doi: 10.1371/journal.pone.0024458 21961036 PMC3178525

[B15] Cai C. Liu S. Liu J. Wen H. Li L. Wang Q. . (2023). Screening and identification of potato StSPS1, a potential crucial gene regulating seed potato vigor. Horticulturae 9, 1–14. doi: 10.3390/horticulturae904051130654563

[B20] Campbell R. Cowan G. Wurzinger B. Ducreux L. J. M. Dessoly J. Guo W. . (2025). GERMIN3 regulates tuber initiation and axillary bud activation by facilitating plasmodesmatal gating. Plant J. 122, 1–15. doi: 10.1111/tpj.70186 40298100 PMC12038876

[B16] Campbell M. A. Gleichsner A. Hilldorfer L. Horvath D. Suttle J. (2012). The sprout inhibitor 1,4-dimethylnaphthalene induces the expression of the cell cycle inhibitors KRP1 and KRP2 in potatoes. Funct. Integr. Genomics 12, 533–541. doi: 10.1007/s10142-011-0257-9 22113341

[B17] Campbell M. A. Gwin C. Tai H. H. Adams R. (2020). Changes in gene expression in potato meristems treated with the sprout suppressor 1,4-dimethylnaphthalene are dependent on tuber age and dormancy status. PloS One 15, 1–13. doi: 10.1371/journal.pone.0235444 32614863 PMC7332019

[B19] Campbell M. Suttle J. Douches D. S. Buell C. R. (2014). Treatment of potato tubers with the synthetic cytokinin 1-(α-ethylbenzyl)-3-nitroguanidine results in rapid termination of endodormancy and induction of transcripts associated with cell proliferation and growth. Funct. Integr. Genomics 14, 789–799. doi: 10.1007/s10142-014-0404-1 25270889

[B18] Campbell M. A. Suttle J. C. Sell Campbell T. W. Sell J. C. Campbeu M. A. (1996). Changes in cell cycle status and expression of p34cdc2 kinase during potato tuber meristem dormancy. Physiol. Plant 98, 743–752. doi: 10.1111/j.1399-3054.1996.tb06680.x40046247

[B21] Chiab N. Kammoun M. Nouri-Ellouz O. Gargouri-Bouzid R. (2023). New potential roles of StDREB1 and VvWRKY2 transcription factors in potato dormancy and sprouting patterns. J. Plant Physiol. 289, 1–8. doi: 10.1016/j.jplph.2023.154077 37683547

[B22] Claassens M. M. J. Vreugdenhil D. (2000). Is dormancy breaking of potato tubers the reverse of tuber initiation? Potato Res. 43, 347–369. doi: 10.1007/BF0236054030311153

[B23] Cools K. Chope G. A. (2026). Re-examining potato dormancy in the context of commercial tuber storage. J. Exp. Bot 1–16. doi: 10.1093/jxb/eraf407 40971991 PMC12794247

[B24] Dai H. Fu M. Yang X. Chen Q. (2016). Ethylene inhibited sprouting of potato tubers by influencing the carbohydrate metabolism pathway. J. Food Sci. Technol. 53, 3166–3174. doi: 10.1007/s13197-016-2290-0 27784911 PMC5055881

[B25] Danieli R. Assouline S. Salam B. B. Vrobel O. Teper-Bamnolker P. Belausov E. . (2023). Chilling induces sugar and ABA accumulation that antagonistically signals for symplastic connection of dormant potato buds. Plant Cell Environ 46, 1–15. doi: 10.1111/pce.14599 37151187

[B26] Debast S. Nunes-Nesi A. Hajirezaei M. R. Hofmann J. Sonnewald U. Fernie A. R. . (2011). Altering trehalose-6-phosphate content in transgenic potato tubers affects tuber growth and alters responsiveness to hormones during sprouting. Plant Physiol. 156, 1754–1771. doi: 10.1104/pp.111.179903 21670224 PMC3149945

[B27] Deng M. Peng J. Zhang J. Ran S. Cai C. Yu L. . (2021). The cysteine-rich peptide snakin-2 negatively regulates tubers sprouting through modulating lignin biosynthesis and h2o2 accumulation in potato. Int. J. Mol. Sci. 22, 1–16. doi: 10.3390/ijms22052287 33669030 PMC7956376

[B28] Destefano-Beltrán L. Knauber D. Huckle L. Suttle J. (2006). Effects of postharvest storage and dormancy status on ABA content, metabolism, and expression of genes involved in ABA biosynthesis and metabolism in potato tuber tissues. Plant Mol. Biol. 61, 687–697. doi: 10.1007/s11103-006-0042-7 16897484

[B29] Devaux A. Goffart J. P. Kromann P. Andrade-Piedra J. Polar V. Hareau G. (2021). The potato of the future: Opportunities and challenges in sustainable agri-food systems. Potato Res. 64, 681–720. doi: 10.1007/s11540-021-09501-4 34334803 PMC8302968

[B30] Di X. Wang Q. Zhang F. Feng H. Wang X. Cai C. (2024). Advances in the modulation of potato tuber dormancy and sprouting. Int. J. Mol. Sci. 25, 1–21. doi: 10.3390/ijms25105078 38791120 PMC11121589

[B31] Dobry E. P. Campbell M. A. (2025). The sprout inhibitor 1,4-dimethylnaphthalene results in common gene expression changes in potato cultivars with varying dormancy profiles. Potato Res. 68, 1023–1048. doi: 10.1007/s11540-024-09772-730311153

[B32] Dogramaci M. Dobry E. P. Fortini E. A. Sarkar D. Eshel D. Campbell M. A. (2024a). Physiological and molecular mechanisms associated with potato tuber dormancy. J. Exp. Bot 75, 1–17. doi: 10.1093/jxb/erae182 38650389 PMC11480654

[B33] Dogramaci M. dos Reis de Oliveira T. Fortini E. A. Sarkar D. Wyatt N. Leonard E. . (2026a). Tissue specific mechanisms of tuber dormancy after 1,4-dimethylnaphthalene treatment in potato. Food. Chemistry: Mol. Sci. 12, 1–19. doi: 10.1016/j.fochms.2026.100376 41777465 PMC12950432

[B34] Dogramaci M. dos Reis de Oliveira T. Fortini E. A. Sarkar D. Wyatt N. Leonard E. . (2026b). Transcriptomics and targeted metabolomics uncover hormonal regulations of potato (Solanum tuberosum L.) tuber dormancy progression under ethylene treatment. Postharvest Biol. Technol. 231, 1–21. doi: 10.1016/j.postharvbio.2025.11387542574925

[B35] Dogramaci M. Sarkar D. Datir S. Finger F. Shetty K. Fugate K. . (2024b). Methyl jasmonate and 1,4-dimethylnaphthalene differentially impact phytohormonal and stress protective pathway regulation involved in potato tuber dormancy. Postharvest Biol. Technol. 213, 1–17. doi: 10.1016/j.postharvbio.2024.11293142574925

[B36] Dun E. A. Germain A. S. Rameau C. Beveridge C. A. (2012). Antagonistic action of strigolactone and cytokinin in bud outgrowth control. Plant Physiol. 158, 487–498. doi: 10.1104/pp.111.186783 22042819 PMC3252097

[B37] Dutta M. Mali S. Raturi V. Zinta G. (2024). Transcriptional and post-transcriptional regulation of tuberization in potato (Solanum tuberosum L.). J. Plant Growth Regul. 43, 1–24. doi: 10.1007/s00344-023-11053-530311153

[B38] European Commission (2019). COMMISSION IMPLEMENTING REGULATION (EU) 2019/989. Off. J. Eur. Union. Available online at: https://eur-lex.europa.eu/legal-content/EN/TXT/HTML/?uri=CELEX:32019R0989 (Accessed August 8, 2025).

[B39] Faivre-Rampant O. Cardle L. Marshall D. Viola R. Taylor M. A. (2004). Changes in gene expression during meristem activation processes in Solanum tuberosum with a focus on the regulation of an auxin response factor gene. J. Exp. Bot. 55, 613–622. doi: 10.1093/jxb/erh075 14966214

[B40] FAOSTAT (2023). Crops and Livestock Products. Available online at: https://www.fao.org/faostat/en/#data/TCL (Accessed October 2023).

[B41] Fernie A. R. Willmitzer L. (2001). Molecular and biochemical triggers of potato tuber development. Plant Physiol. 127, 1459–1465. doi: 10.1104/pp.010764 PMC1540178 11743089

[B42] Ferreira S. J. Senning M. Fischer-Stettler M. Streb S. Ast M. Neuhaus H. E. . (2017). Simultaneous silencing of isoamylases ISA1, ISA2 and ISA3 by multi-target RNAi in potato tubers leads to decreased starch content and an early sprouting phenotype. PloS One 12, 1–21. doi: 10.1371/journal.pone.0181444 28708852 PMC5510849

[B43] Fornara F. Panigrahi K. C. S. Gissot L. Sauerbrunn N. Rühl M. Jarillo J. A. . (2009). Arabidopsis DOF transcription factors act redundantly to reduce CONSTANS expression and are essential for a photoperiodic flowering response. Dev. Cell 17, 75–86. doi: 10.1016/j.devcel.2009.06.015 19619493

[B44] Foukaraki S. G. Cools K. Chope G. A. Terry L. A. (2014). Effect of the transition between ethylene and air storage on postharvest quality in six UK-grown potato cultivars. J. Hortic. Sci. Biotechnol. 89, 599–606. doi: 10.1080/14620316.2014.1151312637339054

[B45] Foukaraki S. G. Cools K. Chope G. A. Terry L. A. (2016a). Impact of ethylene and 1-MCP on sprouting and sugar accumulation in stored potatoes. Postharvest Biol. Technol. 114, 95–103. doi: 10.1016/j.postharvbio.2015.11.01342574925

[B46] Foukaraki S. G. Cools K. Terry L. A. (2016b). Differential effect of ethylene supplementation and inhibition on abscisic acid metabolism of potato (Solanum tuberosum L.) tubers during storage. Postharvest Biol. Technol. 112, 87–94. doi: 10.1016/j.postharvbio.2015.10.00242574925

[B47] Freyre R. Warnke S. Sosinski B. Douches D. S. (1994). Quantitative trait locus analysis of tuber dormancy in diploid potato (Solanum spp.). Theor. Appl. Genet. 89, 474–480. doi: 10.1007/BF00225383 24177897

[B48] Gao Y. Jia L. Hu B. Alva A. Fan M. (2014). Potato stolon and tuber growth influenced by nitrogen form. Plant Prod. Sci. 17, 138–143. doi: 10.1626/pps.17.138

[B49] Gautam S. Scheuring D. C. Koym J. W. Vales M. I. (2024). Assessing heat tolerance in potatoes: Responses to stressful Texas field locations and controlled contrasting greenhouse conditions. Front. Plant Sci. 15, 1–19. doi: 10.3389/fpls.2024.1364244 38803598 PMC11128680

[B50] Guérinier T. Millan L. Crozet P. Oury C. Rey F. Valot B. . (2013). Phosphorylation of p27KIP1 homologs KRP6 and 7 by SNF1-related protein kinase-1 links plant energy homeostasis and cell proliferation. Plant J. 75, 515–525. doi: 10.1111/tpj.12218 23617622

[B51] Guillemette A. M. Casanova G. H. Hamilton J. P. Pokorná E. Dobrev P. I. Motyka V. . (2025). The physiological and molecular responses of potato tuberization to projected future elevated temperatures. Plant Physiol. 197, 1–13. doi: 10.1093/plphys/kiae664 39688842 PMC11683837

[B52] Gutaker R. M. Weiß C. L. Ellis D. Anglin N. L. Knapp S. Luis Fernández-Alonso J. . (2019). The origins and adaptation of European potatoes reconstructed from historical genomes. Nat. Ecol. Evol 3, 1093–1101. doi: 10.1038/s41559-019-0921-3 31235927

[B53] Hancock R. D. Morris W. L. Ducreux L. J. M. Morris J. A. Usman M. Verrall S. R. . (2014). Physiological, biochemical and molecular responses of the potato (Solanum tuberosumL.) plant to moderately elevated temperature. Plant Cell Environ. 37, 439–450. doi: 10.1111/pce.12168 23889235

[B54] Hartmann A. Senning M. Hedden P. Sonnewald U. Sonnewald S. (2011). Reactivation of meristem activity and sprout growth in potato tubers require both cytokinin and gibberellin. Plant Physiol. 155, 776–796. doi: 10.1104/pp.110.168252 21163959 PMC3032466

[B55] Hastilestari B. R. Lorenz J. Reid S. Hofmann J. Pscheidt D. Sonnewald U. . (2018). Deciphering source and sink responses of potato plants (Solanum tuberosum L.) to elevated temperatures. Plant Cell Environ. 41, 2600–2616. doi: 10.1111/pce.13366 29869794

[B56] Hou J. Liu T. Reid S. Zhang H. Peng X. Sun K. . (2019). Silencing of α-amylase StAmy23 in potato tuber leads to delayed sprouting. Plant Physiol. Biochem. 139, 411–418. doi: 10.1016/j.plaphy.2019.03.044 30981157

[B57] Hu Q. Dong Y. Xiang Y. Luo Z. Lu J. Ban Z. . (2025). Exogenous nicotinamide accelerates pyridine nucleotides metabolism and redox homeostasis: A novel strategy for extension of potato dormancy. Postharvest Biol. Technol. 219, 1–12. doi: 10.1016/j.postharvbio.2024.11319842574925

[B58] Hussain H. Mant A. Seale R. Zeeman S. Hinchliffe E. Edwards A. . (2003). Three isoforms of isoamylase contribute different catalytic properties for the debranching of potato glucans. Plant Cell 15, 133–149. doi: 10.1105/tpc.006635 12509527 PMC143484

[B59] Ibañez V. N. Masuelli R. W. Marfil C. F. (2021). Environmentally induced phenotypic plasticity and DNA methylation changes in a wild potato growing in two contrasting Andean experimental gardens. Heredity 126, 50–62. doi: 10.1038/s41437-020-00355-z 32801346 PMC7853039

[B60] Imaizumi T. Schultz T. F. Harmon F. G. Ho L. A. Kay S. A. (2005). FKF1 F-Box protein mediates cyclic degradation of a repressor of CONSTANS in Arabidopsis. Science 309, 293–297. doi: 10.1126/science.1109710 16002617

[B61] Inzé D. De Veylder L. (2006). “ Cell cycle regulation in plant development,” in Annual Review of Genetics, Palo Alto: Annual Reviews. 40, 77–105. doi: 10.1146/annurev.genet.40.110405.090431 17094738

[B62] Jiao A. Gautam S. Pandey J. Scheuring D. C. Koym J. W. Vales M. I. (2026). Genome-wide association studies and genomics-informed breeding for tuber dormancy in tetraploid potatoes. Plant Genome 19, 1–25. doi: 10.1002/tpg2.70205 41710994 PMC12919731

[B63] Jing S. Sun X. Yu L. Wang E. Cheng Z. Liu H. . (2022). Transcription factor StABI5-like 1 binding to the FLOWERING LOCUS T homologs promotes early maturity in potato. Plant Physiol. 189, 1677–1693. doi: 10.1093/plphys/kiac098 35258599 PMC9237700

[B64] Kloosterman B. Abelenda J. A. Gomez M. D. M. C. Oortwijn M. De Boer J. M. Kowitwanich K. . (2013). Naturally occurring allele diversity allows potato cultivation in northern latitudes. Nature 495, 246–250. doi: 10.1038/nature11912 23467094

[B65] Lang G. A. Early J. D. Martin G. C. Darnell R. L. (1987). Endo-, para-, and ecodormancy: physiological terminology and classification for dormancy research. HortScience 22, 371–377. doi: 10.21273/HORTSCI.22.3.371

[B66] Law R. D. Suttle J. C. (2004). Changes in histone H3 and H4 multi-acetylation during natural and forced dormancy break in potato tubers. Physiol. Plant 120, 642–649. doi: 10.1111/j.0031-9317.2004.0273.x 15032826

[B67] Li J. Huang W. Cao H. Xiao G. Zhou J. Xie C. . (2018). Additive and epistatic QTLs underlying the dormancy in a diploid potato population across seven environments. Sci. Hortic. 240, 578–584. doi: 10.1016/j.scienta.2018.06.07142574925

[B69] Li S. Kupriyanovich Y. Wagg C. Zheng F. Hann S. (2023). Water deficit duration affects potato plant growth, yield and tuber quality. Agric. (Switzerland) 13, 1–16. doi: 10.3390/agriculture1310200730654563

[B68] Li J. Tu W. Xiao G. Liu T. Chen H. Tao W. . (2022). Pleiotropic QTL underlying the dormancy and reducing sugar content in potato tubers uncovered by conditional QTL analysis. Potato Res 66, 965–979. doi: 10.1007/s11540-022-09606-430311153

[B70] Lin T. Sharma P. Gonzalez D. H. Viola I. L. Hannapel D. J. (2013). The impact of the long-distance transport of a BEL1-like messenger RNA on development. Plant Physiol. 161, 760–772. doi: 10.1104/pp.112.209429 23221774 PMC3561017

[B74] Liu S. Yang J. Zhang N. Si H. (2025). Genome-wide analysis of non-coding RNA reveals the role of a novel miR319c for tuber dormancy release process in potato. Hortic. Res. 12, 1–19. doi: 10.1093/hr/uhae303 39949878 PMC11822407

[B71] Liu B. Zhang N. Wen Y. Si H. Wang D. (2012). Identification of differentially expressed genes in potato associated with tuber dormancy release. Mol. Biol. Rep. 39, 11277–11287. doi: 10.1007/s11033-012-2037-6 23065212

[B72] Liu B. Zhang N. Zhao S. Chang J. Wang Z. Zhang G. . (2015). Proteomic changes during tuber dormancy release process revealed by iTRAQ quantitative proteomics in potato. Plant Physiol. Biochem. 86, 181–190. doi: 10.1016/j.plaphy.2014.12.003 25514565

[B73] Liu B. Zhao S. Tan F. Zhao H. Wang D. D. Si H. . (2017). Changes in ROS production and antioxidant capacity during tuber sprouting in potato. Food Chem. 237, 205–213. doi: 10.1016/j.foodchem.2017.05.107 28763987

[B75] Martin A. Adam H. Díaz-Mendoza M. Źurczak M. González-Schain N. D. Suárez-López P. (2009). Graft-transmissible induction of potato tuberization by the microRNA miR172. Development 136, 2873–2881. doi: 10.1242/dev.031658 19666819

[B76] Mathura S. R. Sutton F. Rouse-Miller J. Bowrin V. (2024). “ The molecular coordination of tuberization: Current status and future directions,” in Current Opinion in Plant Biology, vol. 82, 1–14. ( Elsevier Ltd). doi: 10.1016/j.pbi.2024.102655 39520793

[B77] Morris W. L. Carmen Alamar M. Lopez-Cobollo R. M. Cañete J. C. Bennett M. Van Der Kaay J. . (2019). A member of the TERMINAL FLOWER 1/CENTRORADIALIS gene family controls sprout growth in potato tubers. J. Exp. Bot. 70, 897–907. doi: 10.1093/jxb/ery387 30395257 PMC6363080

[B78] Navarro C. Abelenda J. A. Cruz-Oró E. Cuéllar C. A. Tamaki S. Silva J. . (2011). Control of flowering and storage organ formation in potato by FLOWERING LOCUS T. Nature 478, 119–122. doi: 10.1038/nature10431 21947007

[B79] Naz R. M. M. Li M. Ramzan S. Li G. Liu J. Cai X. . (2018). QTL mapping for microtuber dormancy and GA3 content in a diploid potato population. Biol. Open 7, 1–7. doi: 10.1242/bio.027375 29212796 PMC5829492

[B80] Pasare S. A. Ducreux L. J. M. Morris W. L. Campbell R. Sharma S. K. Roumeliotis E. . (2013). The role of the potato (Solanum tuberosum) CCD8 gene in stolon and tuber development. New Phytol. 198, 1108–1120. doi: 10.1111/nph.12217 23496288

[B81] Pham G. M. Hamilton J. P. Wood J. C. Burke J. T. Zhao H. Vaillancourt B. . (2020). Construction of a chromosome-scale long-read reference genome assembly for potato. GigaScience 9, 1–11. doi: 10.1093/gigascience/giaa100 32964225 PMC7509475

[B82] Rentzsch S. Podzimska D. Voegele A. Imbeck M. Müller K. Linkies A. . (2012). Dose- and tissue-specific interaction of monoterpenes with the gibberellin-mediated release of potato tuber bud dormancy, sprout growth and induction of α-amylases and β-amylases. Planta 235, 137–151. doi: 10.1007/s00425-011-1501-1 21858448

[B83] Rinne P. L. H. Kaikuranta P. M. Van Schoot C. D. (2001). The shoot apical meristem restores its symplasmic organization during chilling-induced release from dormancy. Plant J. 26, 249–264. doi: 10.1046/j.1365-313X.2001.01022.x 11439114

[B84] Rodríguez-Falcón M. Bou J. Prat S. (2006). “ Seasonal control of tuberization in potato: Conserved elements with the flowering response,” in Annual Review of Plant Biology, (Palo Alto: Annual Reviews). 57, 151–180. doi: 10.1146/annurev.arplant.57.032905.105224 16669759

[B85] Roumeliotis E. Visser R. G. F. Bachem C. W. B. (2012). A crosstalk of auxin and GA during tuber development. Plant Signaling Behav. 7, 1360–1363. doi: 10.4161/psb.21515 22902700 PMC3493427

[B86] Rylski I. Rappaport L. Pratt H. K. (1974). Dual effects of ethylene on potato dormancy and sprout growth. Plant Physiol. 53, 658–662. (Rockville, MD: Oxford University Press on behalf of ASPB). doi: 10.1104/pp.53.4.658 PMC541416 16658762

[B87] Saidi A. Hajibarat Z. (2021). Phytohormones: plant switchers in developmental and growth stages in potato. J. Genet. Eng. Biotechnol. 19, 1–17. doi: 10.1186/s43141-021-00192-5 34142228 PMC8211815

[B88] Senning M. Sonnewald U. Sonnewald S. (2010). Deoxyuridine triphosphatase expression defines the transition from dormant to sprouting potato tuber buds. Mol. Breed. 26, 525–531. doi: 10.1007/s11032-010-9440-230311153

[B89] Shan J. Song W. Zhou J. Wang X. Xie C. Gao X. . (2013). Transcriptome analysis reveals novel genes potentially involved in photoperiodic tuberization in potato. Genomics 102, 388–396. doi: 10.1016/j.ygeno.2013.07.001 23856342

[B90] Sharma P. Lin T. Hannapel D. J. (2016). Targets of the StBEL5 transcription factor include the FT Ortholog StSP6A1[OPEN. Plant Physiol. 170, 310–324. doi: 10.1104/pp.15.01314 26553650 PMC4704582

[B91] Sharma S. K. McLean K. Colgan R. J. Rees D. Young S. Sønderkær M. . (2021). Combining conventional QTL analysis and whole-exome capture-based bulk-segregant analysis provides new genetic insights into tuber sprout elongation and dormancy release in a diploid potato population. Heredity 127, 253–265. doi: 10.1038/s41437-021-00459-0 34331028 PMC8405706

[B93] Śliwka J. Wasilewicz-Flis I. Jakuczun H. Gebhardt C. (2008). Tagging quantitative trait loci for dormancy, tuber shape, regularity of tuber shape, eye depth and flesh colour in diploid potato originated from six Solanum species. Plant Breed. 127, 49–55. doi: 10.1111/j.1439-0523.2008.01420.x40046247

[B92] Simko I. McMurry S. Yang H.-M. Manschot A. Davies P. J. Ewing E. E. (1997). Evidence from polygene mapping for a causal relationship between potato tuber dormancy and abscisic acid content’. Plant Physiol. 115, 1453–1459. doi: 10.1104/pp.115.4.1453 PMC158610 12223876

[B94] Smith A. M. Denyer K. Martin C. (1997). The synthesis of the starch granule. Annu. Rev. Plant Physiol. Plant Mol. Biol. 48, 67–87. (Palo Alto, CA: Annual Reviews). doi: 10.1146/annurev.arplant.48.1.67 15012257

[B95] Sonnewald S. Sonnewald U. (2014). Regulation of potato tuber sprouting. Planta 239 (1), 27–38. (Berlin: Springer Nature / Springer-Verlag). doi: 10.1007/s00425-013-1968-z 24100410

[B96] Stark C. J. Thornton M. Nolte P. (2020). “ Potato production systems,” in Potato Production Systems ( Springer International Publishing). doi: 10.1007/978-3-030-39157-7

[B97] Suttle J. C. (2004). Physiological regulation of potato tuber dormancy. Am. J. Potato Res. 81, 253–262. doi: 10.1007/BF02871767

[B98] Suttle J. C. (2009). Ethylene is not involved in hormone- and Bromoethane-induced dormancy break in Russet Burbank minitubers. Am. J. Potato Res. 86, 278–285. doi: 10.1007/s12230-009-9081-330311153

[B99] Teo C. J. Takahashi K. Shimizu K. Shimamoto K. Taoka K. I. (2017). Potato tuber induction is regulated by interactions between components of a tuberigen complex. Plant Cell Physiol. 58, 365–374. doi: 10.1093/pcp/pcw197 28028166

[B100] Thoma J. Zheljazkov V. D. (2022). Sprout suppressants in potato storage: conventional options and promising essential oils—A review. Sustainability 14 (11), 6382. (Basel: MDPI). doi: 10.3390/su14116382

[B101] Thompson P. G. Haynes F. L. Moll R. H. (1980). Estimation of genetic variance components and heritability for tuber dormancy in diploid potatoes. Am. Potato J. 57, 39–46. doi: 10.1007/BF0286642330311153

[B102] Tosetti R. Waters A. Chope G. A. Cools K. Alamar M. C. McWilliam S. . (2021). New insights into the effects of ethylene on ABA catabolism, sweetening and dormancy in stored potato tubers. Postharvest Biol. Technol. 173, 1–13. doi: 10.1016/j.postharvbio.2020.111420 33658745 PMC7814342

[B103] Van Den Berg J. H. Ewing E. E. Plaisted R. L. Mcmurry S. Bonierbale M. W. (1996). QTL analysis of potato tuber dormancy. Theor. Appl. Genet. 93, 317–324. doi: 10.1007/BF00223171 24162286

[B104] Velappan Y. Signorelli S. Considine M. J. (2017). Cell cycle arrest in plants: What distinguishes quiescence, dormancy and differentiated G1? Ann. Bot. 120, 495–509. doi: 10.1093/aob/mcx082 28981580 PMC5737280

[B105] Vexler L. de la O Leyva-Perez M. Konkolewska A. Clot C. R. Byrne S. Griffin D. . (2024). QTL discovery for agronomic and quality traits in diploid potato clones using PotatoMASH amplicon sequencing. G3: Genes Genomes Genet. 14, 1–21. doi: 10.1093/g3journal/jkae164 39028844 PMC11457057

[B106] Viola R. Pelloux J. Van Der Ploeg A. Gillespie T. Marquis N. Roberts A. G. . (2007). Symplastic connection is required for bud outgrowth following dormancy in potato (Solanum tuberosum L.) tubers. Plant Cell Environ. 30, 973–983. doi: 10.1111/j.1365-3040.2007.01692.x 17617825

[B107] Visse-Mansiaux M. Soyeurt H. Herrera J. M. Torche J. M. Vanderschuren H. Dupuis B. (2022). Prediction of potato sprouting during storage. Field Crops Res. 278. doi: 10.1016/j.fcr.2021.10839642574925

[B108] Visse-Mansiaux M. Tallant M. Brostaux Y. Delaplace P. Vanderschuren H. Dupuis B. (2021). Assessment of pre- and post-harvest anti-sprouting treatments to replace CIPC for potato storage. Postharvest Biol. Technol. 178, 1–10. doi: 10.1016/j.postharvbio.2021.11154042574925

[B109] Wang K. Zhang N. Fu X. Zhang H. Liu S. Pu X. . (2022). StTCP15 regulates potato tuber sprouting by modulating the dynamic balance between abscisic acid and gibberellic acid. Front. Plant Sci. 13, 1–12. doi: 10.3389/fpls.2022.1009552 36186016 PMC9523429

[B110] Ye Y. Jin L. Bian C. Liu J. Guo H. (2024). Monitoring and optimization of potato growth dynamics under different nitrogen forms and rates using UAV RGB imagery. Agronomy 14, 1–14. doi: 10.3390/agronomy1410225730654563

[B111] Zagorščak M. Abdelhakim L. Rodriguez-Granados N. Y. Široká J. Ghatak A. Bleker C. . (2025). Integration of multi-omics data and deep phenotyping provides insights into responses to single and combined abiotic stress in potato. Plant Physiol. 197, 1–19. doi: 10.1093/plphys/kiaf126 40173380 PMC12012603

[B112] Zakaria M. Hossain M. M. Mian M. A. K. Hossain T. Sultana N. (2007). Effect of nitrogen and potassium on *in vitro* tuberization of potato. Plant Tissue Culture Biotechnol. 17, 79–85. doi: 10.3329/ptcb.v17i1.112440208441

[B116] Zhang J. He N. Liu C. Xu L. Chen Z. Li Y. . (2019). Variation and evolution of C:N ratio among different organs enable plants to adapt to N-limited environments. Global Change Biol. 26, 2534–2543. doi: 10.1111/gcb.14973 31873968

[B117] Zhang M. Jian H. Shang L. Wang K. Wen S. Li Z. . (2024). Transcriptome analysis reveals novel genes potentially involved in tuberization in potato. Plants 13, 1–20. doi: 10.3390/plants13060795 38592791 PMC10975680

[B114] Zhang H. Liu X. Nie B. Song B. Du P. Liu S. . (2022). Nitrogen management can inhibit or induce the sprouting of potato tubers: Consequences of regulation tuberization. Postharvest Biol. Technol. 183, 1–12. doi: 10.1016/j.postharvbio.2021.11172242574925

[B115] Zhang H. Liu X. Song B. Nie B. Wei Zhang Zhao Z. (2020). Effect of excessive nitrogen on levels of amino acids and sugars, and differential response to post-harvest cold storage in potato (Solanum tuberosum L.) tubers. Plant Physiol. Biochem. 157, 38–46. doi: 10.1016/j.plaphy.2020.09.040 33069979

[B113] Zhang G. Tang R. Niu S. Si H. Yang Q. Rajora O. P. . (2021). Heat-stress-induced sprouting and differential gene expression in growing potato tubers: Comparative transcriptomics with that induced by postharvest sprouting. Hortic. Res. 8, 1–18. doi: 10.1038/s41438-021-00680-2 34654802 PMC8519922

[B119] Zheng X. Li M. Zhang X. Chen J. Ge X. Li S. . (2024). Unraveling the mechanism of potato (Solanum tuberosum L.) tuber sprouting using transcriptome and metabolome analyses. Front. Plant Sci. 14, 1–14. doi: 10.3389/fpls.2023.1300067 38250446 PMC10796687

[B118] Zheng H. Wang Y. Zhao J. Shi X. Ma Z. Fan M. (2018). Tuber formation as influenced by the C: N ratio in potato plants. J. Plant Nutr. Soil Sci. 181, 686–693. doi: 10.1002/jpln.20170057142587375

[B120] Zhou T. Song B. Liu T. Shen Y. Dong L. Jing S. . (2019). Phytochrome F plays critical roles in potato photoperiodic tuberization. Plant J. 98, 42–54. doi: 10.1111/tpj.14198 30552774

[B121] Zwanenburg B. Blanco-Ania D. (2018). Strigolactones: New plant hormones in the spotlight. J. Exp. Bot. 69, 2205–2218. doi: 10.1093/jxb/erx487 29385517

