# Supplementary material for: Environmental, genetic and physiological factors governing potato endodormancy: from tuber development to endodormancy break
Source: Front Plant Sci. 2026 Oct 12;17:1858517. doi: 10.3389/fpls.2026.1858517 (PMC13697010; doi:10.3389/fpls.2026.1858517)
Supplement: Supplementary file 1 [file Table1.docx]

Table 1. Comprehensive overview of different regulatory components involved in different stages of potato tubers from stolon growth to tuber sprouting.

| **Stage of development** | **Name** | **Regulatory components** | **Function and crosstalk** | **Upregulated or downregulated** | **Cite** |
| --- | --- | --- | --- | --- | --- |
| Stolon growth | *StARF8* | Gene | Auxin‑responsive transcription factor regulating stolon growth and promoting tuberization | Upregulated | Sharma et al. (2016) |
| Stolon growth | StPIN1 | Transporter protein | Auxin efflux carrier regulating auxin distribution during stolon growth | Upregulated | Cho et al. (2015) |
| Stolon growth and initiation | StCDPK1 | Protein kinase | Calcium‑dependent kinase transmitting Ca²⁺ signals to regulate tuberization and phosphorylating auxin transporters | Upregulated | Santin et al. (2016) |
| Stolon growth and initiation | *StLOG1* | Gene | Gene involved in cytokinin activation and biosynthesis promote tuber initiation and tuberization, with cytokinin biosynthesis specifically regulated by BEL5. | Upregulated | Bhogale et al. (2014); Sharma et al. (2016) |
| Stolon growth and initiation | StPIN4 | Transporter protein | Auxin transporter involved in tuber formation and interacting with ARF8 and CDPK1 to regulate auxin flow during tuber initiation | Upregulated | Santin et al. (2016) |
| Tuber induction | *StCOL1* | Gene | Photoperiod‑dependent repressor of tuberization under long‑day conditions | Upregulated in long-day conditions | Abelenda et al. (2016) |
| Tuber induction | StPHYB | Photoreceptor protein | Red/far‑red photoreceptor modulating photoperiodic control of tuberization through interaction with CO and SP6A | Upregulated | Rodríguez-Falcón et al. (2006) |
| Tuber induction | *StPIF3* | Gene | Temperature‑responsive transcription factor suppressing StSP6A under high temperatures and interacting with TOC1 | Upregulated | Dutta et al. (2024) |
| Tuber induction | *StTOC1* | Gene | Circadian clock regulator interacting with PIF3 to modulate photoperiodic tuberization | Upregulated | Dutta et al. (2024); Wang et al. (2023) |
| Tuber induction and initiation | *StCDF1* | Gene | DOF transcription factor repressing StCO to permit StSP6A activation and promoting early tuberization | Upregulated | Kloosterman et al. (2013) |
| Tuber initiation | *POTH1* | Gene | Homeobox transcription factor promoting tuber formation | Upregulated | Chen et al. (2003); Rosin et al. (2003) |
| Tuber initiation | St14‑3‑3 | Protein | Regulatory protein forming part of the tuberigen activation complex with SP6A and FDL1 | Upregulated | Teo et al. (2017) |
| Tuber initiation | *StABL1* | Gene | Transcription factor interacting with FT‑like proteins to promote early maturity and tuber initiation | Upregulated | Jing et al. (2022) |
| Tuber initiation | *StAFB4* | Gene | Auxin receptor modulating auxin‑dependent tuberization | Upregulated | Kolachevskaya et al. (2017) |
| Tuber initiation | *StAFB6* | Gene | Auxin receptor regulating auxin response during tuber initiation | Upregulated | Kolachevskaya et al. (2017) |
| Tuber initiation | StAOC | Enzyme | Cyclase enzyme in jasmonic acid biosynthesis promoting tuberization | Upregulated | Liu et al. (2025) |
| Tuber initiation | StAOS1 | Enzyme | Enzyme in the jasmonic acid biosynthesis pathway acting upstream of LOX3 | Upregulated | Begum et al. (2022); Chen & Setter (2021) |
| Tuber initiation | *StBEL11* | Gene | Transcription factor suppressing tuberization by antagonizing BEL5 and repressing growth‑promoting genes | Upregulated under high temperatures | Ghate et al. (2017) |
| Tuber initiation | *StBEL29* | Gene | Transcription factor suppressing tuberization similarly to BEL11 | Upregulated under high temperatures | Ghate et al. (2017) |
| Tuber initiation | *StBEL5* | Gene | Mobile RNA signal regulating tuberization by interacting with GA and cytokinin pathways, acting upstream of StCDF1, and interacting with POTH1, StSP6A, and SWEET11 to modulate growth and carbon allocation | Upregulated | Chen et al. (2003); Banerjee et al. (2006) |
| Tuber initiation | *StBMI1* | Gene | Chromatin‑modifying regulator suppressing tuberization through histone modification | Upregulated | Kumar et al. (2020) |
| Tuber initiation | *StBRC1B* | Gene | Transcription factor regulating bud dormancy and tuber formation by inhibiting SP6A transport | Upregulated | Nicolas et al. (2022) |
| Tuber initiation | *StCDF1.2* | Gene | Allelic variant of CDF1 contributing to early tuberization by repressing CO and enhancing SP6A | Upregulated | Salaria et al. (2020); Kloosterman et al. (2013) |
| Tuber initiation | *StCDF1.3* | Gene | Allelic variant associated with early maturity through enhanced SP6A expression | Upregulated | Salaria et al. (2020); Kloosterman et al. (2013) |
| Tuber initiation | *StE(z)2* | Gene | Histone methyltransferase regulating chromatin state; reduced expression enhances tuber yield | Downregulated | Kumar et al. (2021) |
| Tuber initiation | *StERF3* | Gene | Ethylene‑responsive transcription factor enhancing tuberization | Upregulated | Zhang et al. (2024) |
| Tuber initiation | *StFTL1* | Gene | FT‑like transcription factor regulating tuberization through interactions with ABL1, SP3D, and SP6A | Upregulated | Jing et al. (2023) |
| Tuber initiation | StGA20ox1 | Enzyme | Gibberellin biosynthesis enzyme inhibiting tuberization by promoting GA accumulation | Upregulated | Bou-Torrent et al. (2011) |
| Tuber initiation | StGA3ox1 | Enzyme | Gibberellin biosynthesis enzymes are downregulated during tuber initiation but enhance sprout elongation, reflecting GA’s dual role in dormancy release and growth. | Downregulated during tuber initiation. Upregulated during sprouting | Bou-Torrent et al. (2011) |
| Tuber initiation | StGA3ox2 | Enzyme | Gibberellin biosynthesis enzymes suppress tuberization by antagonizing GA2ox1, while also contributing to GA accumulation during sprouting. | Downregulated during tuber initiation. Upregulated during sprouting | Bou-Torrent et al. (2011) |
| Tuber initiation | *StKNOX* | Gene | Homeobox transcription factor interacting with BEL5 in tuberization signaling | Upregulated | Hannapel et al. (2017) |
| Tuber initiation | StLOX3 | Enzyme | Lipoxygenase enzyme involved in jasmonic acid biosynthesis during tuber initiation | Upregulated | Begum et al. (2022); Chen & Setter (2021) |
| Tuber initiation | *StMYC2* | Gene | Transcription factor mediating JA responses in tuberization | Upregulated | Begum et al. (2022); Chen & Setter (2021) |
| Tuber initiation | *StPOTH1* | Gene | Homeobox transcription factor interacting with BEL5 to regulate tuberization | Upregulated | Tengli et al. (2022); Hastilestari et al. (2018) |
| Tuber initiation | StPTB1 | RNA‑binding protein | RNA‑binding protein involved in tuberization and targeted by phased siRNAs | Upregulated | Cho et al. (2015) |
| Tuber initiation | *StRAP1* | Gene | Negative regulator of tuberization repressing BEL5 | Upregulated | Martin et al. (2009); Bhogale et al. (2014) |
| Tuber initiation | *StSP3D* | Gene | FT‑like gene contributing to tuber formation through interactions with ABL1, SP6A, and FTL1 | Upregulated | Jing et al. (2022); Jing et al. (2023) |
| Tuber initiation | *StSP5G* | Gene | FT‑like repressor of tuberization suppressing SP6A expression, especially under long days and heat | Upregulated in long-day conditions and under high temperatures | Abelenda et al. (2016) |
| Tuber initiation | StSP6A | Protein | Mobile tuberization signal promoting tuber initiation; interacts with ABL1, SP3D, FDL1, BEL5, CDF1, BRC1B, and SWEET11, forms the tuberigen activation complex, and is regulated by photoperiod and temperature | Upregulated. Downregulated under high temperatures | Sharma et al. (2016) |
| Tuber initiation | StTIR1 | Receptor protein | Auxin receptor regulating auxin response genes during tuber initiation | Upregulated | Kolachevskaya et al. (2017) |
| Tuber initiation and development | StJAZ1 | Protein | Jasmonate signaling repressor modulating tuber initiation and interacting with GA and ABA pathways | Upregulated | Begum et al. (2022); Chen & Setter (2021) |
| Tuber initiation and development | StJAZ1‑like | Protein | Jasmonate signaling regulator modulating tuber initiation and interacting with GA and ABA pathways | Upregulated | Begum et al. (2022) |
| Tuber initiation and development | StSUT1 | Transporter protein | Sucrose transporter regulating source–sink balance and directing sugars toward tuber formation; sensitive to high‑temperature suppression | Upregulated during tuber initiation and development. Downregulated during tuber initiation and development under high temperatures. Upregulated during sprouting | Kühn et al. (2003) |
| Tuber initiation and dormancy | *StABI5* | Gene | ABA‑responsive transcription factor regulating tuber dormancy and interacting with GA and ABA pathways | Upregulated | Jing et al. (2022) |
| Tuber initiation, dormancy and sprouting | StGA2ox1 | Enzyme | Gibberellin catabolism enzymes reduce GA levels to regulate tuberization, sprouting, and dormancy; they are primarily controlled by SP6A/StSP6A and influenced by jasmonic acid (JA), abscisic acid (ABA), photoperiod, and other hormonal signaling pathways, with some enzymes specifically maintaining dormancy while others promote tuberization and sprout growth. | Upregulated | Kloosterman et al. (2007) |
| Tuber initiation and sprouting | *StCEN* | Gene | Negative regulator of tuberization that competes with SP6A also functions as a regulator of sprout growth and dormancy, interacting with gibberellin (GA) and cytokinin signaling pathways. | Upregulated during tuber initiation. Downregulated during sprouting | Zhang et al. (2020b); Morris et al. (2019) |
| Tuber development | *StAGAMOUS‑like* | Gene | Transcription factor regulating floral and tuber developmental pathways | Upregulated | Jing et al. (2022) |
| Tuber development | *StAGL8* | Gene | MADS‑box transcription factor regulating tuber initiation and development | Upregulated | Kumar et al. (2021); Kolachevskaya et al. (2019) |
| Tuber development | StHSP20 | Protein | Heat shock protein protecting against heat stress during tuber development | Upregulated | Zhao et al. (2018) |
| Tuber development | StmiR156 | microRNA | MicroRNA-mediated post-transcriptional regulator that promotes tuberization by repressing SPL genes and targeting BRC1B | Upregulated | Bhogale et al. (2014) |
| Tuber development | StmiR172 | microRNA | MicroRNA promoting tuberization through RAP1 repression and enhanced StBEL5 activity | Upregulated | Jing et al. (2022) |
| Tuber development | StPOTLX1 | Enzyme | Lipoxygenase involved in auxin‑related regulation of tuber growth | Upregulated | Kolachevskaya et al. (2019) |
| Tuber development | *StPOTM1* | Gene | MADS‑box transcription factor downstream of SP6A and BEL5, regulating tuber identity; downregulated under high temperatures | Downregulated under high temperatures | Ševčíková et al. (2017); Tengli et al. (2022); Zhang et al. (2024) |
| Tuber development and dormancy | StPYR1‑like | Receptor protein | ABA receptors contributing to tuberization and dormancy maintenance, being regulated respectively by ABL1 and ABI5. | Upregulated | Jing et al. (2022); Chen & Setter (2021) |
| Tuber development and sprouting | StSWEET11 | Transporter protein | Sucrose transporter interacting with SP6A to regulate carbon partitioning | Upregulated | Abelenda et al. (2019) |
| Tuber dormancy | ACS (1‑aminocyclopropane‑1‑carboxylate synthase) | Enzyme | Enzyme involved in ethylene biosynthesis; downregulated during dormancy due to ethylene inhibition (e.g., 1‑MCP) | Downregulated | Tosetti et al. (2021) |
| Tuber dormancy | Ethylene receptor homolog (ERH) | Receptor protein | Ethylene receptor participating in dormancy regulation; expression increases with ethylene and decreases with 1‑MCP | Upregulated (ethylene), Downregulated (1-MCP) | Tosetti et al. (2021) |
| Tuber dormancy | Snakin‑2 (StSN2) | Peptide | Cysteine‑rich peptide involved in brassinosteroid signaling, modulating lignin biosynthesis and H₂O₂ accumulation during dormancy | Upregulated | Li et al. (2022b); Deng et al. (2021) |
| Tuber dormancy | Starch Phosphorylase (STP) | Enzyme | Enzyme catalyzing reversible starch breakdown and synthesis during dormancy | Upregulated | Sergeeva et al. (2012); Dai et al. (2016) |
| Tuber dormancy | StGAPC1 | Enzyme | Glycolytic enzyme contributing to energy metabolism and ROS balance during dormancy | Upregulated | Li et al. (2022b) |
| Tuber dormancy | StPYL4 | Receptor protein | ABA receptor involved in dormancy regulation and controlled by ABI5 | Upregulated | Begum et al. (2022) |
| Tuber dormancy, dormancy break and sprouting | StNCED1 | Enzyme | ABA biosynthesis enzymes regulating ABA levels in cortex tissues, being downregulated during dormancy progression and sprouting, and inhibited by nitric oxide to reduce ABA and promote sprouting. | Downregulated during tuber dormancy and dormancy break. Upregulated during sprouting | Destefano-Beltrán et al. (2006); Wang et al. (2020) |
| Tuber dormancy and sprouting | AGPase | Enzyme | Key enzyme in starch biosynthesis regulating the balance between starch synthesis and degradation during dormancy | Upregulated | Sergeeva et al. (2012) |
| Dormancy break | ACO (1‑aminocyclopropane‑1‑carboxylate oxidase) | Enzyme | Enzyme in ethylene biosynthesis regulating ethylene‑induced dormancy break; downregulated by 1‑MCP | Upregulated (ethylene), Downregulated (1-MCP) | Tosetti et al. (2021) |
| Dormancy break | *RD22* | Gene | ABA‑responsive gene involved in dehydration and dormancy regulation; downregulated during dormancy break | Downregulated | Campbell et al. (2008) |
| Dormancy break | SLP | Protease | Protease involved in dormancy maintenance; downregulated during transition to sprouting | Downregulated | Zhang et al. (2021); Liu et al. (2017) |
| Dormancy break | SnRK1 | Kinase | Energy‑sensing kinase regulating metabolic reprogramming; its reduction promotes dormancy release and sprouting | Downregulated during dormancy break. Upregulated during sprouting | Debast et al, 2011; Danieli et al. (2023) |
| Dormancy break | StNOS‑IP | Protein | Protein enhancing nitric oxide production, promoting ABA degradation and dormancy release | Upregulated | Wang et al. (2020) |
| Dormancy break | StNR | Enzyme | Nitrate reductase contributing to nitric oxide production and ABA reduction during dormancy release | Upregulated | Wang et al. (2020); Shaikh et al. (2025) |
| Dormancy break | StVInv | Enzyme | Vacuolar invertase regulating sugar accumulation and energy balance during dormancy break | Upregulated | Danieli et al. (2023) |
| Dormancy break | α‑Amylase | Enzyme | Enzyme involved in starch hydrolysis during dormancy break | Upregulated | Hou et al, 2019 |
| Dormancy break | β‑Amylase | Enzyme | Enzyme converting starch into maltose during dormancy release | Upregulated | Hou et al, 2019 |
| Dormancy break and sprouting | *DOG1* | Gene | Dormancy‑associated gene whose downregulation marks dormancy release and sprouting onset | Downregulated | Zhang et al. (2021) |
| Dormancy break and sprouting | GA20ox1 | Enzyme | GA biosynthesis enzyme promoting sprouting during dormancy release | Upregulated | Chen et al, 2004 |
| Dormancy break and sprouting | SNF4 | Kinase | Mitochondrial kinase affecting energy sensing pathways linked to dormancy release and sprouting | Downregulated in dormancy break. Upregulated during sprouting | Zhang et al. (2021) |
| Dormancy break and sprouting | StCYP707A1 | Enzyme | ABA catabolism enzymes promote abscisic acid degradation, thereby facilitating dormancy release and stimulating sprouting by lowering ABA levels. | Upregulated during dormancy break. Downregulated during sprouting | Liu et al. (2017b) |
| Sprouting | *ARF1* | Gene | Auxin‑responsive transcription factor possibly involved in cell division and early sprouting | Upregulated | Liu et al. (2012) |
| Sprouting | *ARF6* | Gene | Auxin‑responsive transcription factor promoting sprout elongation | Upregulated | Faivre-Rampant et al. (2004) |
| Sprouting | *ARG7* | Gene | Gene involved in arginine metabolism and nitrogen signaling; possibly linked to dormancy release | Upregulated | Bisognin et al. (2018) |
| Sprouting | BAM | Enzyme | β‑Amylase enzyme hydrolyzing starch into maltose during sprouting and energy mobilization | Upregulated | Zhang et al. (2014) |
| Sprouting | BAP (6‑benzylaminopurine) | Plant growth regulator | Synthetic cytokinin promoting cell division, shoot proliferation, and dormancy release | Upregulated | Moletsane et al. (2022) |
| Sprouting | CAT1 | Enzyme | Catalase enzyme detoxifying hydrogen peroxide; involved in oxidative stress response during sprouting | Downregulated | Rojas-Beltran et al. (2000) |
| Sprouting | CAT2 | Enzyme | Catalase isoform contributing to ROS balance and stress tolerance in tuber tissues | Downregulated | Rojas-Beltran et al. (2000) |
| Sprouting | CCD8 | Enzyme | Carotenoid cleavage dioxygenase involved in strigolactone biosynthesis and shoot branching regulation | Downregulated | Pasare et al. (2013) |
| Sprouting | CISZOG | Enzyme | Cytokinin‑specific O‑glucosyltransferase regulating cytokinin homeostasis by conjugation | Differently expressed | Zhang et al. (2023) |
| Sprouting | *CYCD1* | Gene | Cyclin D gene promoting cell cycle progression during sprout activation | Downregulated | Campbell et al. (2012) |
| Sprouting | *CYCD2* | Gene | Cyclin D gene regulating G1/S transition in meristematic cells during sprouting | Downregulated | Campbell et al. (2012) |
| Sprouting | *CYCD3* | Gene | Cyclin D gene enhancing cell division and shoot growth during sprouting | Downregulated | Campbell et al. (2012) |
| Sprouting | CYP735A | Enzyme | Enzyme involved in trans‑zeatin biosynthesis, contributing to cytokinin signaling during growth | Differently expressed | Zhang et al. (2023) |
| Sprouting | *ERF1* | Gene | Ethylene‑responsive transcription factor modulating ABA and GA pathways during sprouting | Upregulated | Dobry & Campbell (2025) |
| Sprouting | GA2ox2 | Enzyme | Gibberellin catabolism enzyme reducing active GA levels to regulate sprouting and elongation | Upregulated | Dogramaci et al. (2024) |
| Sprouting | GA3 | Plant growth regulator | Gibberellic acid promoting sprout elongation and dormancy release | Upregulated | Moletsane et al. (2022); Naz et al. (2018) |
| Sprouting | GA3ox1 | Enzyme | GA biosynthesis enzyme enhancing sprout elongation | Upregulated | Kolachevskaya et al. (2017) |
| Sprouting | GA3ox2 | Enzyme | GA biosynthesis enzyme contributing to GA accumulation during sprouting | Upregulated | Kolachevskaya et al. (2017) |
| Sprouting | GAD2 | Enzyme | Glutamate decarboxylase involved in GABA biosynthesis; may modulate stress and sprouting signals | Upregulated | Bisognin et al. (2018) |
| Sprouting | *GH3.5* | Gene | Auxin‑responsive gene conjugating IAA to amino acids; regulates auxin homeostasis during sprouting | Upregulated | Bisognin et al. (2018) |
| Sprouting | *GH3.6* | Gene | Auxin‑responsive gene modulating IAA levels and stress responses during sprouting | Upregulated | Bisognin et al. (2018) |
| Sprouting | HXK1 | Enzyme | Hexokinase involved in sugar sensing and energy metabolism during sprout activation | Upregulated | Tiessen et al. (2003) |
| Sprouting | *IAA15* | Gene | Auxin‑induced gene regulating cell elongation and sprout development | Differently expressed | Zhang et al. (2023) |
| Sprouting | *IAA16* | Gene | Auxin‑responsive gene possibly involved in early sprout growth and hormonal crosstalk | Upregulated | Zhang et al. (2021) |
| Sprouting | *IAA22* | Gene | Auxin‑responsive gene contributing to sprout elongation and auxin signaling | Differently expressed | Zhang et al. (2023) |
| Sprouting | *IPT* | Gene | Isopentenyltransferase catalyzing cytokinin biosynthesis and promoting sprouting | Upregulated | Kolachevskaya et al. (2017) |
| Sprouting | ISA1, ISA2, ISA3 | Enzyme complex | Enzymes involved in starch debranching and remodeling during sprouting | Downregulated | Hussain et al. (2003); Ferreira et al. (2017) |
| Sprouting | *KRP1* | Gene | Kip‑related protein regulating cell cycle progression and meristem activity during sprouting | Upregulated | Campbell et al. (2012) |
| Sprouting | *KRP2* | Gene | Cell cycle inhibitor modulating shoot meristem activity and sprout growth | Upregulated | Campbell et al. (2012) |
| Sprouting | LOX1 | Enzyme | Lipoxygenase involved in jasmonic acid biosynthesis and stress signaling during sprouting | Upregulated | Kolomiets et al. (2001) |
| Sprouting | *PGSC0003DMT400004597* | Gene | Putative gene possibly involved in hormonal regulation or stress response during sprouting | Upregulated | Zhang et al. (2021) |
| Sprouting | *PGSC0003DMT400013627* | Gene | Predicted gene possibly contributing to sugar metabolism or dormancy release | Upregulated | Zhang et al. (2021) |
| Sprouting | *PGSC0003DMT400020589* | Gene | Uncharacterized gene possibly involved in sprout activation or hormonal signaling | Upregulated | Zhang et al. (2021) |
| Sprouting | *PK1N1* | Gene | Protein kinase possibly regulating hormonal signaling and sprout development | Upregulated | Gong et al. (2021) |
| Sprouting | POTLX‑1 | Enzyme | Lipoxygenase involved in auxin‑related regulation of tuber growth and sprouting | Upregulated | Kolomiets et al. (2001) |
| Sprouting | *POTM1‑1* | Gene | MADS‑box transcription factor downstream of SP6A and BEL5, regulating tuber identity and sprouting | Upregulated | Kumar et al. (2021) |
| Sprouting | PPase | Enzyme | Pyrophosphatase regulating phosphate metabolism and energy balance during sprouting | Upregulated | Si et al. (2016) |
| Sprouting | *SAUR50* | Gene | Small auxin‑up RNA gene promoting cell expansion and sprout elongation | Differently expressed | Zhang et al. (2023) |
| Sprouting | Snakin‑2 | Peptide | Cysteine‑rich peptide involved in brassinosteroid signaling and ROS modulation during sprouting | Downregulated | Deng et al. (2021) |
| Sprouting | SPS | Enzyme | Sucrose phosphate synthase, involved in carbohydrate metabolism | Upregulated | Cai et al. (2023) |
| Sprouting | StAmy23 | Enzyme | α‑Amylase isoform hydrolyzing starch into sugars during sprouting and early growth | Upregulated | Hou et al. (2019). |
| Sprouting | STARCH PHOSPHORYLASE | Enzyme | Enzyme catalyzing reversible starch breakdown and synthesis during dormancy and sprouting | Upregulated | Dai et al. (2016) |
| Sprouting | StCYP707A2 | Enzyme | ABA 8′‑hydroxylase contributing to ABA degradation and dormancy release | Upregulated | Destefano-Beltrán et al. (2006) |
| Sprouting | *StDREB1* | Gene | Drought‑responsive transcription factor possibly involved in stress signaling during sprouting | Upregulated | Chiab et al. (2023) |
| Sprouting | StGA2ox | Enzyme | Gibberellin catabolism enzyme reducing GA levels to regulate sprouting and elongation | Downregulated | Kloosterman et al. (2007) |
| Sprouting | StHSP26 | Protein | Heat shock protein protecting sprouting tissues from thermal stress | Upregulated | Tang et al. (2020) |
| Sprouting | *StIAA2* | Gene | Auxin‑responsive gene regulating cell elongation and sprout development | Upregulated | Kloosterman et al. (2006) |
| Sprouting | *StNS2* | Gene | Putative gene possibly involved in nitrogen signaling or sprouting regulation | Downregulated | Deng et al. (2021) |
| Sprouting | StPP2Ac2b | Protein | Protein phosphatase involved in ABA signaling and dormancy regulation | Upregulated | Muñiz García et al. (2022) |
| Sprouting | StSUT4 | Transporter protein | Sucrose transporter modulating sugar allocation during sprouting and tuber development | Upregulated | Garg et al. (2021) |
| Sprouting | *StTCP15* | Gene | TCP transcription factor regulating cell proliferation and sprout elongation | Upregulated | Wang et al. (2022); Che et al. (2022) |
| Sprouting | *TCP14* | Gene | TCP transcription factor modulating shoot development and sprouting | Upregulated | Wang et al. (2022) |
| Sprouting | TUBBY | Protein | Putative signaling protein possibly involved in hormonal crosstalk and sprout regulation | Upregulated | Campbell et al. (2008) |
| Sprouting | UGT85A1 | Enzyme | UDP‑glucosyltransferase conjugating hormones like cytokinins; regulates hormonal homeostasis during sprouting | Differently expressed | Zhang et al. (2023) |
| Sprouting | *VvWRKY2* | Gene | WRKY transcription factor from grapevine; possibly involved in stress signaling and sprouting when expressed in potato | Upregulated | Chiab et al. (2023) |
| Sprouting | Zeatin | Plant growth regulator | Natural cytokinin promoting cell division, shoot growth, and dormancy | Upregulated | Moletsane et al. (2022) |

Abelenda, J. A., Bergonzi, S., Oortwijn, M., Sonnewald, S., Du, M., Visser, R. G. F., Sonnewald, U., & Bachem, C. W. B. (2019). Source-Sink Regulation Is Mediated by Interaction of an FT Homolog with a SWEET Protein in Potato. *Current Biology*, *29*(7), 1178-1186.e6. https://doi.org/10.1016/j.cub.2019.02.018

Abelenda, J. A., Cruz-Oró, E., Franco-Zorrilla, J. M., & Prat, S. (2016). Potato StCONSTANS-like1 Suppresses Storage Organ Formation by Directly Activating the FT-like StSP5G Repressor. *Current Biology*, *26*(7), 872–881. https://doi.org/10.1016/j.cub.2016.01.066

Banerjee, A. K., Chatterjee, M., Yu, Y., Suh, S. G., Miller, W. A., & Hannapel, D. J. (2006). Dynamics of a mobile RNA of potato involved in a long-distance signaling pathway. *Plant Cell*, *18*(12), 3443–3457. https://doi.org/10.1105/tpc.106.042473

Begum, S., Jing, S., Yu, L., Sun, X., Wang, E., Abu Kawochar, M., Qin, J., Liu, J., & Song, B. (2022). Modulation of JA signalling reveals the influence of StJAZ1-like on tuber initiation and tuber bulking in potato. *Plant Journal*, *109*(4), 952–964. https://doi.org/10.1111/tpj.15606

Bhogale, S., Mahajan, A. S., Natarajan, B., Rajabhoj, M., Thulasiram, H. V., & Banerjee, A. K. (2014). MicroRNA156: A potential graft-transmissible microrna that modulates plant architecture and tuberization in Solanum tuberosum ssp. andigena. *Plant Physiology*, *164*(2), 1011–1027. https://doi.org/10.1104/pp.113.230714

Bisognin, D. A., Manrique-Carpintero, N. C., & Douches, D. S. (2018). QTL Analysis of Tuber Dormancy and Sprouting in Potato. *American Journal of Potato Research*, *95*(4), 374–382. https://doi.org/10.1007/s12230-018-9638-0

Bou-Torrent, J., Martínez-García, J. F., García-Martínez, J. L., & Prat, S. (2011). Gibberellin a 1 metabolism contributes to the control of photoperiod-mediated tuberization in potato. *PLoS ONE*, *6*(9). https://doi.org/10.1371/journal.pone.0024458

Cai, C., Liu, S., Liu, J., Wen, H., Li, L., Wang, Q., Li, L., & Wang, X. (2023). Screening and Identification of Potato StSPS1, a Potential Crucial Gene Regulating Seed Potato Vigor. *Horticulturae*, *9*(4). https://doi.org/10.3390/horticulturae9040511

Campbell, M. A., Gleichsner, A., Hilldorfer, L., Horvath, D., & Suttle, J. (2012). The sprout inhibitor 1,4-dimethylnaphthalene induces the expression of the cell cycle inhibitors KRP1 and KRP2 in potatoes. *Functional and Integrative Genomics*, *12*(3), 533–541. https://doi.org/10.1007/s10142-011-0257-9

Campbell, M., Segear, E., Beers, L., Knauber, D., & Suttle, J. (2008). Dormancy in potato tuber meristems: Chemically induced cessation in dormancy matches the natural process based on transcript profiles. *Functional and Integrative Genomics*, *8*(4), 317–328. https://doi.org/10.1007/s10142-008-0079-6

Che, Y., Liao, Y. Q., Fu, X., Yue, Y., Zhang, N., & Si, H. J. (2022). Regulation of StTCP15 gene expression and tuber dormancy characteristics of potato by gibberellic acid, abscisic acid, and low temperature. *Biologia Plantarum*, *66*, 96–102. https://doi.org/10.32615/bp.2021.065

Chen, C. T., & Setter, T. L. (2021). Role of tuber developmental processes in response of potato to high temperature and elevated co2. *Plants*, *10*(5). https://doi.org/10.3390/plants10050871

Chen, H., Rosin, F. M., Prat, S., & Hannapel, D. J. (2003). Interacting transcription factors from the three-amino acid loop extension superclass regulate tuber formation. *Plant Physiology*, *132*(3), 1391–1404. https://doi.org/10.1104/pp.103.022434

Chiab, N., Kammoun, M., Nouri-Ellouz, O., & Gargouri-Bouzid, R. (2023). New potential roles of StDREB1 and VvWRKY2 transcription factors in potato dormancy and sprouting patterns. *Journal of Plant Physiology*, *289*. https://doi.org/10.1016/j.jplph.2023.154077

Cho, S. K., Sharma, P., Butler, N. M., Kang, I. H., Shah, S., Rao, A. G., & Hannapel, D. J. (2015). Polypyrimidine tract-binding proteins of potato mediate tuberization through an interaction with StBEL5 RNA. *Journal of Experimental Botany*, *66*(21), 6835–6847. https://doi.org/10.1093/jxb/erv389

Dai, H., Fu, M., Yang, X., & Chen, Q. (2016). Ethylene inhibited sprouting of potato tubers by influencing the carbohydrate metabolism pathway. *Journal of Food Science and Technology*, *53*(8), 3166–3174. https://doi.org/10.1007/s13197-016-2290-0

Danieli, R., Assouline, S., Salam, B. B., Vrobel, O., Teper-Bamnolker, P., Belausov, E., Granot, D., Tarkowski, P., & Eshel, D. (2023). Chilling induces sugar and ABA accumulation that antagonistically signals for symplastic connection of dormant potato buds. *Plant Cell and Environment*. https://doi.org/10.1111/pce.14599

Deng, M., Peng, J., Zhang, J., Ran, S., Cai, C., Yu, L., Ni, S., Huang, X., Li, L., & Wang, X. (2021). The cysteine-rich peptide snakin-2 negatively regulates tubers sprouting through modulating lignin biosynthesis and h2o2 accumulation in potato. *International Journal of Molecular Sciences*, *22*(5), 1–16. https://doi.org/10.3390/ijms22052287

Destefano-Beltrán, L., Knauber, D., Huckle, L., & Suttle, J. (2006). Effects of postharvest storage and dormancy status on ABA content, metabolism, and expression of genes involved in ABA biosynthesis and metabolism in potato tuber tissues. *Plant Molecular Biology*, *61*, 687–697. https://doi.org/10.1007/s11103

Dobry, E. P., & Campbell, M. A. (2025). The Sprout Inhibitor 1,4-Dimethylnaphthalene Results in Common Gene Expression Changes in Potato Cultivars with Varying Dormancy Profiles. *Potato Research*, *68*(2), 1023–1048. https://doi.org/10.1007/s11540-024-09772-7

Dogramaci, M., Dobry, E. P., Fortini, E. A., Sarkar, D., Eshel, D., & Campbell, M. A. (2024). Physiological and molecular mechanisms associated with potato tuber dormancy. *Journal of Experimental Botany*. https://doi.org/10.1093/jxb/erae182

Dutta, M., Mali, S., Raturi, V., & Zinta, G. (2024). Transcriptional and Post-transcriptional Regulation of Tuberization in Potato (Solanum tuberosum L.). In *Journal of Plant Growth Regulation* (Vol. 43, Number 1, pp. 1–24). Springer. https://doi.org/10.1007/s00344-023-11053-5

Faivre-Rampant, O., Cardle, L., Marshall, D., Viola, R., & Taylor, M. A. (2004). Changes in gene expression during meristem activation processes in Solanum tuberosum with a focus on the regulation of an auxin response factor gene. *Journal of Experimental Botany*, *55*(397), 613–622. https://doi.org/10.1093/jxb/erh075

Ferreira, S. J., Senning, M., Fischer-Stettler, M., Streb, S., Ast, M., Neuhaus, H. E., Zeeman, S. C., Sonnewald, S., & Sonnewald, U. (2017). Simultaneous silencing of isoamylases ISA1, ISA2 and ISA3 by multi-target RNAi in potato tubers leads to decreased starch content and an early sprouting phenotype. *PLoS ONE*, *12*(7). https://doi.org/10.1371/journal.pone.0181444

Garg, V., Hackel, A., & Kühn, C. (2021). Expression level of mature miR172 in wild type and STSUT4-silenced plants of solanum tuberosum is sucrose-dependent. *International Journal of Molecular Sciences*, *22*(3), 1–11. https://doi.org/10.3390/ijms22031455

Ghate, T., Sharma, P., Kondhare, K., Hannapel, D., & Banerjee, A. (2017). The mobile RNAs, StBEL11 and StBEL29, suppress growth of tubers in potato. *Plant Mol Biol*, 563–578. https://doi.org/10.1007/s11103-016-0582-4

Gong, H. L., Dusengemungu, L., Igiraneza, C., & Rukundo, P. (2021). Molecular regulation of potato tuber dormancy and sprouting: a mini-review. In *Plant Biotechnology Reports* (Vol. 15, Number 4, pp. 417–434). Springer. https://doi.org/10.1007/s11816-021-00689-y

Hannapel, D. J., Sharma, P., Lin, T., & Banerjee, A. K. (2017). The multiple signals that control tuber formation. In *Plant Physiology* (Vol. 174, Number 2, pp. 845–856). American Society of Plant Biologists. https://doi.org/10.1104/pp.17.00272

Hastilestari, B. R., Lorenz, J., Reid, S., Hofmann, J., Pscheidt, D., Sonnewald, U., & Sonnewald, S. (2018). Deciphering source and sink responses of potato plants (Solanum tuberosum L.) to elevated temperatures. *Plant Cell and Environment*, *41*(11), 2600–2616. https://doi.org/10.1111/pce.13366

Hou, J., Liu, T., Reid, S., Zhang, H., Peng, X., Sun, K., Du, J., Sonnewald, U., & Song, B. (2019). Silencing of α-amylase StAmy23 in potato tuber leads to delayed sprouting. *Plant Physiology and Biochemistry*, *139*, 411–418. https://doi.org/10.1016/j.plaphy.2019.03.044

Hussain, H., Mant, A., Seale, R., Zeeman, S., Hinchliffe, E., Edwards, A., Hylton, C., Bornemann, S., Smith, A. M., Martin, C., & Bustos, R. (2003). Three isoforms of isoamylase contribute different catalytic properties for the debranching of potato glucans. *Plant Cell*, *15*(1), 133–149. https://doi.org/10.1105/tpc.006635

Jing, S., Jiang, P., Sun, X., Yu, L., Wang, E., Qin, J., Zhang, F., Prat, S., & Song, B. (2023). Long-distance control of potato storage organ formation by SELF PRUNING 3D and FLOWERING LOCUS T-like 1. *Plant Communications*. https://doi.org/10.1016/j.xplc.2023.100547

Jing, S., Sun, X., Yu, L., Wang, E., Cheng, Z., Liu, H., Jiang, P., Qin, J., Begum, S., & Song, B. (2022). Transcription factor StABI5-like 1 binding to the FLOWERING LOCUS T homologs promotes early maturity in potato. *Plant Physiology*, *189*(3), 1677–1693. https://doi.org/10.1093/plphys/kiac098

Kloosterman, B., Abelenda, J. A., Gomez, M. D. M. C., Oortwijn, M., De Boer, J. M., Kowitwanich, K., Horvath, B. M., Van Eck, H. J., Smaczniak, C., Prat, S., Visser, R. G. F., & Bachem, C. W. B. (2013). Naturally occurring allele diversity allows potato cultivation in northern latitudes. *Nature*, *495*(7440), 246–250. https://doi.org/10.1038/nature11912

Kloosterman, B., Navarro, C., Bijsterbosch, G., Lange, T., Prat, S., Visser, R., & Bachem, C. W. B. (2007). StGA2ox1 is induced prior to stolon swelling and controls GA levels during potato tuber development. *The Plant Journal*, *52*, 362–373. https://doi.org/10.1111/j.1365-313X.2007.03245.x

Kloosterman, B., Visser, R. G. F., & Bachem, C. W. B. (2006). Isolation and characterization of a novel potato Auxin/Indole-3-Acetic Acid family member (StIAA2) that is involved in petiole hyponasty and shoot morphogenesis. *Plant Physiology and Biochemistry*, *44*(11–12), 766–775. https://doi.org/10.1016/j.plaphy.2006.10.026

Kolachevskaya, O. O., Lomin, S. N., Arkhipov, D. V., & Romanov, G. A. (2019). Auxins in potato: molecular aspects and emerging roles in tuber formation and stress resistance. In *Plant Cell Reports* (Vol. 38, Number 6, pp. 681–698). Springer Verlag. https://doi.org/10.1007/s00299-019-02395-0

Kolachevskaya, O. O., Sergeeva, L. I., Floková, K., Getman, I. A., Lomin, S. N., Alekseeva, V. V., Rukavtsova, E. B., Buryanov, Y. I., & Romanov, G. A. (2017). Auxin synthesis gene tms1 driven by tuber-specific promoter alters hormonal status of transgenic potato plants and their responses to exogenous phytohormones. *Plant Cell Reports*, *36*(3), 419–435. https://doi.org/10.1007/s00299-016-2091-y

Kolomiets, M. V, Hannapel, D. J., Chen, H., Tymeson, M., & Gladon, R. J. (2001). Lipoxygenase Is Involved in the Control of Potato Tuber Development. In *The Plant Cell* (Vol. 13). www.plantcell.org

Kühn, C., Hajirezaei, M. R., Fernie, A. R., Roessner-Tunali, U., Czechowski, T., Hirner, B., & Frommer, W. B. (2003). The sucrose transporter StSUT1 localizes to sieve elements in potato tuber phloem and influences tuber physiology and development. *Plant Physiology*, *131*(1), 102–113. https://doi.org/10.1104/pp.011676

Kumar, A., Kondhare, K. R., Malankar, N. N., & Banerjee, A. K. (2021). The Polycomb group methyltransferase StE(z)2 and deposition of H3K27me3 and H3K4me3 regulate the expression of tuberization genes in potato. *Journal of Experimental Botany*, *72*(2), 426–444. https://doi.org/10.1093/jxb/eraa468

Kumar, V., Aulakh, C. S., Kaur, J., & Chawla, N. (2020). Nutrition management effects on productivity and tuber quality of potato (Solanum tuberosum). *Indian Journal of Agronomy*, *65*(3), 337–343. https://www.researchgate.net/publication/352438172

Li, L., Lyu, C., Chen, J., Lu, Y., Yang, S., Ni, S., Zheng, S., Yu, L., Wang, X., Wang, Q., & Lu, L. (2022). Snakin-2 interacts with cytosolic glyceraldehyde-3-phosphate dehydrogenase 1 to inhibit sprout growth in potato tubers. *Horticulture Research*, *9*. https://doi.org/10.1093/hr/uhab060

Liu, B., Zhang, N., Wen, Y., Si, H., & Wang, D. (2012). Identification of differentially expressed genes in potato associated with tuber dormancy release. *Molecular Biology Reports*, *39*(12), 11277–11287. https://doi.org/10.1007/s11033-012-2037-6

Liu, L., Zhang, R. J., Zhu, W. J., Liu, X. R., Shi, K., Chen, M., & Yang, Q. (2017). Inhibitory effect of StCYP707A1 gene on tuberization in transgenic potato. *Plant Biotechnology Reports*, *11*(4), 219–228. https://doi.org/10.1007/s11816-017-0442-y

Liu, S., Yang, J., Zhang, N., & Si, H. (2025). Genome-wide analysis of non-coding RNA reveals the role of a novel miR319c for tuber dormancy release process in potato. *Horticulture Research*, *12*(2). https://doi.org/10.1093/hr/uhae303

Martin, A., Adam, H., Díaz-Mendoza, M., Źurczak, M., González-Schain, N. D., & Suárez-López, P. (2009). Graft-transmissible induction of potato tuberization by the microRNA miR172. *Development*, *136*(17), 2873–2881. https://doi.org/10.1242/dev.031658

Moletsane, M. S., Kimurto, P. K., & Oyoo, M. E. (2022). Effect of Presprouting Plant Growth Regulators and Natural Materials on Dormancy, Growth, and Yield of Potatoes (Solanum tuberosum L.). *Advances in Agriculture*, *2022*. https://doi.org/10.1155/2022/9932606

Morris, W. L., Carmen Alamar, M., Lopez-Cobollo, R. M., Cañete, J. C., Bennett, M., Van Der Kaay, J., Stevens, J., Sharma, S. K., McLean, K., Thompson, A. J., Terry, L. A., Turnbull, C. G. N., Bryan, G. J., & Taylor, M. A. (2019). A member of the TERMINAL FLOWER 1/CENTRORADIALIS gene family controls sprout growth in potato tubers. *Journal of Experimental Botany*, *70*(3), 897–907. https://doi.org/10.1093/jxb/ery387

Muñiz García, M. N., Cortelezzi, J. I., & Capiati, D. A. (2022). The protein phosphatase 2A catalytic subunit StPP2Ac2b is involved in the control of potato tuber sprouting and source-sink balance in tubers and sprouts. *Journal of Experimental Botany*, *73*(19), 6784–6799. https://doi.org/10.1093/jxb/erac326

Naz, R. M. M., Li, M., Ramzan, S., Li, G., Liu, J., Cai, X., & Xie, C. (2018). QTL mapping for microtuber dormancy and GA3 content in a diploid potato population. *Biology Open*, *7*(1). https://doi.org/10.1242/bio.027375

Nicolas, M., Torres-Pérez, R., Wahl, V., Cruz-Oró, E., Rodríguez-Buey, M. L., Zamarreño, A. M., Martín-Jouve, B., García-Mina, J. M., Oliveros, J. C., Prat, S., & Cubas, P. (2022). Spatial control of potato tuberization by the TCP transcription factor BRANCHED1b. *Nature Plants*, *8*(3), 281–294. https://doi.org/10.1038/s41477-022-01112-2

Pasare, S. A., Ducreux, L. J. M., Morris, W. L., Campbell, R., Sharma, S. K., Roumeliotis, E., Kohlen, W., van der Krol, S., Bramley, P. M., Roberts, A. G., Fraser, P. D., & Taylor, M. A. (2013). The role of the potato (Solanum tuberosum) CCD8 gene in stolon and tuber development. *New Phytologist*, *198*(4), 1108–1120. https://doi.org/10.1111/nph.12217

Rodríguez-Falcón, M., Bou, J., & Prat, S. (2006). Seasonal control of tuberization in potato: Conserved elements with the flowering response. In *Annual Review of Plant Biology* (Vol. 57, pp. 151–180). Annual Reviews Inc. https://doi.org/10.1146/annurev.arplant.57.032905.105224

Rojas-Beltran, J. A., Dejaeghere, F., Abd, M., Kotb, A., & Du, P. (2000). Expression and activity of antioxidant enzymes during potato tuber dormancy. In *Potato Research* (Vol. 43).

Rosin, F. M., Hart, J. K., Horner, H. T., Davies, P. J., & Hannapel, D. J. (2003). Overexpression of a knotted-like homeobox gene of potato alters vegetative development by decreasing gibberellin accumulation. *Plant Physiology*, *132*(1), 106–117. https://doi.org/10.1104/pp.102.015560

Salaria, N., Siddappa, S., Thakur, K., Tomar, M., Goutam, U., Sharma, N., Sood, S., Bhardwaj, V., & Singh, B. (2020). Solanum tuberosum (CYCLING DOF FACTOR) CDF1.2 allele: A candidate gene for developing earliness in potato. *South African Journal of Botany*, *132*, 242–248. https://doi.org/10.1016/j.sajb.2020.05.008

Santin, F., Bhogale, S., Fantino, E., Grandellis, C., Banerjee, A. K., & Ulloa, R. M. (2016). Solanum tuberosum StCDPK1 is regulated by miR390 at the posttranscriptional level and phosphorylates the auxin efflux carrier StPIN4 in vitro, a potential downstream target in potato development (261, Tran.). *Physiologia Plantarum*, 244. https://doi.org/10.1002/ppl.12517

Sergeeva, L. I., Claassens, M. M. J., Jamar, D. C. L., van der Plas, L. H. W., & Vreugdenhil, D. (2012). Starch-related enzymes during potato tuber dormancy and sprouting. *Russian Journal of Plant Physiology*, *59*(4), 556–564. https://doi.org/10.1134/S1021443712040115

Ševčíková, H., Mašková, P., Tarkowská, D., Mašek, T., & Lipavská, H. (2017). Carbohydrates and gibberellins relationship in potato tuberization. *Journal of Plant Physiology*, *214*, 53–63. https://doi.org/10.1016/j.jplph.2017.04.003

Shaikh, M. A., Ramírez-Gonzales, L., Franco-Zorrilla, J. M., Steiner, E., Oortwijn, M., Bachem, C. W. B., & Prat, S. (2025). StCDF1: A ‘jack of all trades’ clock output with a central role in regulating potato nitrate reduction activity. *New Phytologist*, *245*(1), 282–298. https://doi.org/10.1111/nph.20186

Sharma, P., Lin, T., & Hannapel, D. J. (2016). Targets of the StBEL5 transcription factor include the FT Ortholog StSP6A1[OPEN]. *Plant Physiology*, *170*(1), 310–324. https://doi.org/10.1104/pp.15.01314

Si, H., Zhang, C., Zhang, N., Wen, Y., & Wang, D. (2016). Control of potato tuber dormancy and sprouting by expression of sense and antisense genes of pyrophosphatase in potato. *Acta Physiol Plant*, *38*, 38–69. https://doi.org/10.1007/s11738-016-2089-7

Tang, R., Gupta, S., Niu, S., Li, X.-Q., Yang, Q., Chen, G., Zhu, W. J., & Haroon, M. (2020). Transcriptome analysis of heat stress response genes in potato leaves. *Molecular Biology Reports*, 4311–4321. https://doi.org/10.1007/s11033-020-05485-5

Tengli, S., Narasimhamurthy, S. T., Koppad, A., Govind, G., & Raju, B. M. (2022). Shoot and root zone temperatures are critical in bidirectional regulation of tuberization in potato. *Environmental and Experimental Botany*, *201*. https://doi.org/10.1016/j.envexpbot.2022.104936

Teo, C. J., Takahashi, K., Shimizu, K., Shimamoto, K., & Taoka, K. I. (2017). Potato tuber induction is regulated by interactions between components of a tuberigen complex. *Plant and Cell Physiology*, *58*(2), 365–374. https://doi.org/10.1093/pcp/pcw197

Tiessen, A., Prescha, K., Branscheid, A., Palacios, N., McKibbin, R., Halford, N., & Geigenberger, P. (2003). Evidence that SNF1-related kinase and hexokinase are involved in sugar-signalling pathways. *The Plant Journal*, *35*, 490–500. https://doi.org/10.1046/j.1365-313X.2003.01823.x

Tosetti, R., Waters, A., Chope, G. A., Cools, K., Alamar, M. C., McWilliam, S., Thompson, A. J., & Terry, L. A. (2021). New insights into the effects of ethylene on ABA catabolism, sweetening and dormancy in stored potato tubers. *Postharvest Biology and Technology*, *173*. https://doi.org/10.1016/j.postharvbio.2020.111420

Wang, E., Zhou, T., Jing, S., Dong, L., Sun, X., Fan, Y., Shen, Y., Liu, T., & Song, B. (2023). Leaves and stolons transcriptomic analysis provide insight into the role of phytochrome F in potato flowering and tuberization. *Plant Journal*, *113*(2), 402–415. https://doi.org/10.1111/tpj.16056

Wang, K., Zhang, N., Fu, X., Zhang, H., Liu, S., Pu, X., Wang, X., & Si, H. (2022). StTCP15 regulates potato tuber sprouting by modulating the dynamic balance between abscisic acid and gibberellic acid. *Frontiers in Plant Science*, *13*. https://doi.org/10.3389/fpls.2022.1009552

Wang, Z., Ma, R., Zhao, M., Wang, F., Zhang, N., & Si, H. (2020). NO and ABA Interaction Regulates Tuber Dormancy and Sprouting in Potato. *Frontiers in Plant Science*, *11*. https://doi.org/10.3389/fpls.2020.00311

Zhang, G., Tang, R., Niu, S., Si, H., Yang, Q., Rajora, O. P., & Li, X. Q. (2021). Heat-stress-induced sprouting and differential gene expression in growing potato tubers: Comparative transcriptomics with that induced by postharvest sprouting. *Horticulture Research*, *8*(1). https://doi.org/10.1038/s41438-021-00680-2

Zhang, H., Hou, J., Liu, J., Xie, C., & Song, B. (2014). Amylase Analysis in Potato Starch Degradation During Cold Storage and Sprouting. *Potato Research*, 47–58. https://doi.org/10.1007/s11540-014-9252-6

Zhang, M., Jian, H., Shang, L., Wang, K., Wen, S., Li, Z., Liu, R., Jia, L., Huang, Z., & Lyu, D. (2024). Transcriptome Analysis Reveals Novel Genes Potentially Involved in Tuberization in Potato. *Plants*, *13*(6). https://doi.org/10.3390/plants13060795

Zhang, X., Campbell, R., Ducreux, L. J. M., Morris, J., Hedley, P. E., Mellado-Ortega, E., Roberts, A. G., Stephens, J., Bryan, G. J., Torrance, L., Chapman, S., Prat, S., & Taylor, M. A. (2020). TERMINAL FLOWER‐1/CENTRORADIALIS inhibits tuberisation via protein interaction with the tuberigen activation complex. *The Plant Journal*, *103*, 2263–2278. https://doi.org/10.1111/tpj.14898

Zhang, X., Fujino, K., & Shimura, H. (2023). Transcriptomic Analyses Reveal the Role of Cytokinin and the Nodal Stem in Microtuber Sprouting in Potato (Solanum tuberosum L.). *International Journal of Molecular Sciences*, *24*, 2–22. https://doi.org/10.3390/ijms242417534

Zhao, P., Wang, D. D., Wang, R., Kong, N., Zhang, C., Yang, C., Wu, W., Ma, H., & Chen, Q. (2018). Genome-wide analysis of the potato Hsp20 gene family: identification, genomic organization and expression profiles in response to heat stress. *BMC Genomics*, 19–61. https://doi.org/10.1186/s12864-018-4443-1
